# Expression of a large LINE-1-driven antisense RNA is linked to epigenetic silencing of the metastasis suppressor gene *TFPI-2* in cancer

**DOI:** 10.1093/nar/gkt438

**Published:** 2013-05-23

**Authors:** Hazel A. Cruickshanks, Natasha Vafadar-Isfahani, Donncha S. Dunican, Andy Lee, Duncan Sproul, Jonathan N. Lund, Richard R. Meehan, Cristina Tufarelli

**Affiliations:** ^1^Wolfson Centre for Stem Cells, Tissue Engineering and Modelling (STEM), School of Clinical Sciences, University of Nottingham, Centre for Biomedical Sciences, Nottingham NG7 2RD, UK, ^2^School of Graduate Entry Medicine, University of Nottingham, Royal Derby Hospital, Derby DE22 3DT, UK, ^3^MRC Human Genetics Unit, Institute of Genetics and Molecular Medicine, University of Edinburgh, Edinburgh EH4 2XU, UK, ^4^Breakthrough Research Unit, University of Edinburgh, Edinburgh EH4 2XU, UK and ^5^Centre for Genetics and Genomics, University of Nottingham, Queens Medical Centre, Nottingham NG7 2RD, UK

## Abstract

LINE-1 retrotransposons are abundant repetitive elements of viral origin, which in normal cells are kept quiescent through epigenetic mechanisms. Activation of LINE-1 occurs frequently in cancer and can enable LINE-1 mobilization but also has retrotransposition-independent consequences. We previously reported that in cancer, aberrantly active LINE-1 promoters can drive transcription of flanking unique sequences giving rise to LINE-1 chimeric transcripts (LCTs). Here, we show that one such LCT, LCT13, is a large transcript (>300 kb) running antisense to the metastasis-suppressor gene *TFPI-2*. We have modelled antisense RNA expression at *TFPI-2* in transgenic mouse embryonic stem (ES) cells and demonstrate that antisense RNA induces silencing and deposition of repressive histone modifications implying a causal link. Consistent with this, LCT13 expression in breast and colon cancer cell lines is associated with silencing and repressive chromatin at *TFPI-2*. Furthermore, we detected LCT13 transcripts in 56% of colorectal tumours exhibiting reduced *TFPI-2* expression. Our findings implicate activation of LINE-1 elements in subsequent epigenetic remodelling of surrounding genes, thus hinting a novel retrotransposition-independent role for LINE-1 elements in malignancy.

## INTRODUCTION

Tumourigenesis is accompanied by multiple genetic and epigenetic changes that promote cell proliferation and escape from growth arrest (1). Epigenetic alterations include disruption of DNA methylation patterns, changes in histone modifications and nucleosome positioning, ultimately contributing to modified gene expression (2). Changes to DNA methylation in cancer are well characterized, with both genome-wide hypomethylation at repetitive sequences such as retrotransposons and hypermethylation of CpG island (CGI) genes present within the same malignant cell type (3,4). The molecular mechanisms underlying these epigenetic aberrancies remain elusive; but it is possible that a relationship exists between genome-wide changes in DNA modifications and alterations in gene expression (4–7).

Decreased methylation of LINE-1 (L1) retroelements is frequently observed in cancer (8), and examples of direct correlations between the degree of hypomethylation and the severity of the disease have been shown (9). Commensurate with this, hypomethylation-induced activation of L1s has been proposed to promote cancer progression and genomic instability (10–12) due to retrotransposition events (13–15), and indeed, recent evidence confirms the presence of new somatic L1 insertions in the tumours of patients with lung and colon cancer (16,17).

A role in genomic instability and chromosomal rearrangements is apparent when considering evolutionary young and intact L1 elements; however, the majority of L1 elements are older and retrotransposition deficient due to truncations or accumulated mutations (18). There are approximately 500 000 truncated L1 copies in the human genome and about 7000 potential full-length copies; the majority of which have accumulated mutations that prevent them from active retrotransposition while retaining intact promoter sequences (18,19). Therefore, several retrotransposition incompetent elements remain capable of transcription, and there is evidence that this may contribute to the transcriptome of mammalian cells (20).

L1 promoters are bidirectional, containing a sense promoter responsible for transcription within the L1 element and an antisense promoter (L1-ASP) that can drive transcription of adjacent regions giving rise to transcripts composed partly of L1 and partly of genomic sequence (LINE-1 chimeric transcripts [LCTs]) (21,22). It is recognized that cancers have a high degree of hypomethylation at repetitive elements (23–25) and that L1s can promote transcription of cellular genes as a consequence of ASP activity (21,26,27). Recent evidence suggests the existence of a causal link between aberrant activation of individual L1-ASP promoters and cancer development and progression. For example, the hypomethylated L1-ASP of an intronic LINE-1 has been shown to act as an alternative promoter driving expression of a truncated isoform of the oncogene cMET (28–30). Our group has also shown that hypomethylation-induced activation of L1-ASP promoters can drive transcription of cancer-specific LCTs transcribed in the same (sense) or opposite (antisense) orientation with respect to neighbouring genes (22). Interestingly, the majority of naturally occurring non-coding antisense transcripts, which can have both activating and silencing effects (31), have been found to initiate at transposable elements (26) suggesting an additional mechanism by which LCTs could affect gene expression. However, given the abundance of L1 elements within the genome, the full extent to which aberrant transcriptional activation of L1 promoters occurs and its relevance to cancer development and progression are not thoroughly understood. Here, we aimed to determine whether aberrant activation of L1-ASP has the potential to trigger epigenetic silencing of linked cancer genes through mechanisms similar to those previously described for antisense RNAs in normal development and disease (32–35).

## MATERIALS AND METHODS

### Ethics statement

Ethical approval for the study was obtained from the Derbyshire Research Ethics Committee to collect colorectal tumour and matched normal tissue from patients who underwent surgical cancer resection at the Royal Derby Hospital, Derby, UK.

### Tissue samples

All breast samples in this study were purchased from AMS biotechnology and Ambion. Matched normal and tumour colorectal tissues were donated by consenting patients undergoing surgical cancer resection at the Royal Derby Hospital, Derby, UK.

### Cell culture

All cell lines used in the study were validated by STR profiling (Biosynthesys, USA). 

MCF-7, HCC-1954, and T47D cell lines were grown in RPMI 1640 medium and HCT116 and CaCo-2 in Modified Eagle's Media (MEM) and Dulbecco's Modified Eagle Medium (DMEM), respectively (Gibco, Invitrogen). All cultures were supplemented with 10% foetal bovine serum and 100 µg/ml penicillin-streptomycin (Gibco, Invitrogen) and incubated at 37°C with 5% CO_2_. The polymerase chain reaction (PCR) primers and reaction conditions used for the study are described in Supplementary Data, Tables S1–S5.

### Strand-specific RT–PCR

Total RNA was extracted from cells using Total RNA Isolation reagent (Sigma) and was treated with DNaseI (Invitrogen). Reverse transcription reactions on 1 µg of DNaseI-treated total RNA were performed using superscript II (Invitrogen). PCR reactions were performed on 1 µl of first-strand cDNA by mixing 1× Thermo Pol *Taq* buffer (New England Biolabs), 0.2 mM dNTPs (Amersham), 0.25 µM forward primer, 0.25 µM reverse primer, and 1.25 U *Taq* polymerase (New England Biolabs) and incubated at 95°C for 5 min followed by 30 cycles of 95°C for 1 min, appropriate annealing temperature for 1 min and 72°C for 1 min with a final incubation at 72°C for 10 min.

### Bisulphite sequencing

One microgram of genomic DNA was digested with an appropriate restriction enzyme and bisulphite treated as published (36). Only clones with >99% conversion of non-CpG cytosines to uracil were used for the analysis. Analysis of sequencing data was performed using the BiQ analyser (37).

### Real-time RT–PCR

Two microlitres of reverse-transcription (RT) reaction (see above) was used as a template for real-time PCR using inventoried Taq Man probes (Applied Biosystems) as per manufacturer’s recommendations using an ABI 7500 fast PCR machine. The Taqman probe for LCT13b was designed using ABI software and custom made. Expression data were analysed using GraphPad Prism 5.

### 3′ RACE

Total RNA was reverse transcribed as described using oligo AUAP-dT (an oligodT primer containing the AUAP adaptor sequence at its 5′ end) to prime the reactions. Two rounds of nested PCR were performed using primer AUAP in combination with primer 0118 or 0124 for primary and secondary PCR, respectively. PCR products were cloned using the pGEM-T Easy vector system (Promega), and Sanger sequencing was outsourced to Source Biosciences.

### Generation of constructs and ES cell clones

For pTFPI-2as and pTFPI-2pa constructs, a 4.93-kb human genomic DNA fragment including the full-length *TFPI-2* gene obtained by long-range PCR (Expand Long PCR kit, Roche) on human genomic DNA with primers HC63f and HC63g and was cloned into the BamHI and KpnI sites of pcDNA3 (Invitrogen) and pcDNA3p(A)for, respectively. pcDNA3p(A)for was derived from pcDNA3 by cloning a 262 bp BGHp(A) fragment, obtained by PCR on pcDNA3 with primers Hind-p(A)-for and Hind-p(A)-rev, into the HindIII site of pcDNA3. Prior to electroporation constructs were linearized with ScaI.

E14Tg2a ES cells were maintained undifferentiated in gelatin-treated flasks in High Glucose DMEM (Invitrogen) supplemented with 10^3^ U/ml leukaemia inhibitory factor (LIF; Esgro, Millipore), 18% foetal calf serum (SLI), 10 µM β-mercaptoethanol (Sigma) and 1× each of sodium pyruvate and non-essential amino acids (Invitrogen). Stable ES cell clones were obtained by electroporation of 1–2 × 10^7^ ES cells with 50 µg of purified linearized construct fragment containing the neomycin resistance gene. G418-resistant colonies were analysed by PCR for construct integration. ES cells differentiation was carried out as previously described (34).

### Chromatin immunoprecipitation

Soluble chromatin from mouse ES cells was isolated using micrococcal nuclease as described (38). Native chromatin immunoprecipitations were carried out on chromatin pre-cleared in Protein G sepharose (GE Healthcare) in block buffer (0.5% BSA in PBS) following published protocols (39) with Protein G pre-bound antibodies (5–10 µg): &nbsp;α-H3K9me3 (Abcam #8898), αH4K20me3 (Abcam #9053) and rabbit IgG serum (Santa Cruz Biotechnology) as control. Immunoprecipitated DNA was purified using QiaQuick columns (Qiagen). quantitative PCR (qPCR) was performed using Roche multiprobe with primer pairs in the CGI and intron 2 of *TFPI-2*, and normalized to ActB.

Chromatin immunoprecipitations (ChIPs) for cancer cell lines were performed on 0.4% formaldehyde X-linked chromatin using the EZ-Magna ChIP A kit (Millipore) according to manufacturer’s recommendations using the following antibodies: α-H3K9me3 (Diagenode pAb-056-050), α-H4K20me3 (Diagenode pAb-057-050), α-H3K27me3 (Diagenode pAb-069-050), α-H3K4me3 (Diagenode pAb-003-050) and the rabbit IgG provided. qPCR was performed using custom Taqman assays for TFPI-2prom, TFPI-2ex2 and *APRT* 3′UTR. For the *GAPDH* promoter, primers were provided with the EZ-Magna ChIP A kit and used with PowerSYBR (AB). 

Primers used for qPCR are listed in Supplementary Tables S4 and S5.

### Statistics

Statistical analyses were performed with the GraphPad software using the Wilcoxon matched-pair signed rank test.

## RESULTS

### Expression of LCT13 correlates with presence of TFPI-2 antisense transcripts and TFPI-2 downregulation

**Figure 1.**
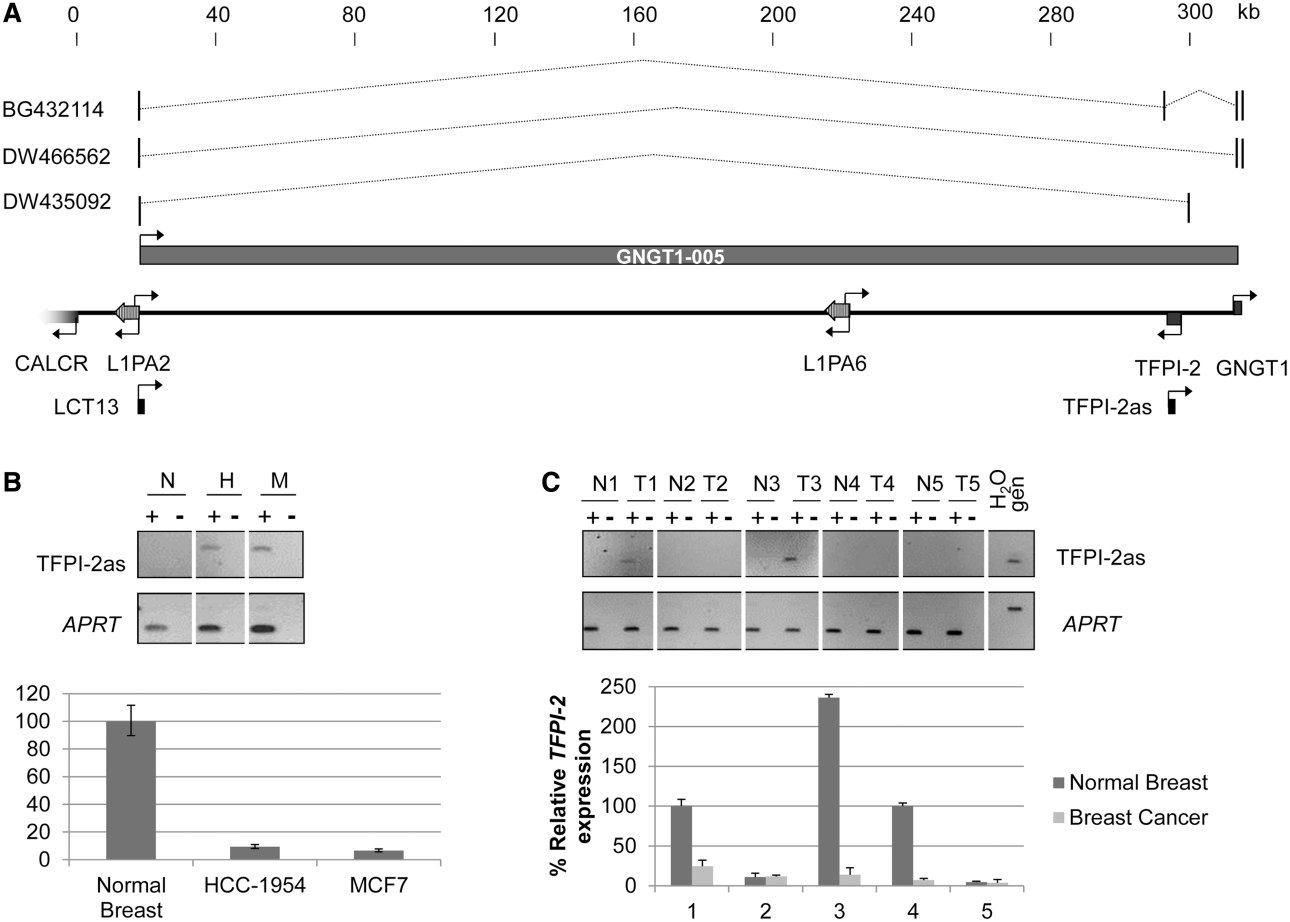
Correlated expression of LCT13 and TFPI-2as transcripts in breast cancer cells. (**A**) Schematic diagram of a 300-kb region of chromosome 7q21.3 including LCT13 and the *TFPI-2* gene. Scale is kilobase and indicates the position from the centromere with the value of 0 arbitrarily assigned to the TSS of *CALCR*. Genes (5′ segment of *CALCR*, *TFPI-2* and *GNGT1*) are indicated as gray arrows. Two LINE-1 elements are present in the region (L1PA2 and L1PA6). Transcriptional orientations are indicated by arrows. LCT13 is a previously identified transcript shown to initiate at an L1ASP by 5′ RACE (22). TFPI-2as is the fragment analysed by strand-specific RT–PCR to test for the presence of *TFPI-2* antisense RNAs. Displayed are the three spliced ESTs isolated from kidney (BG432114) and liver (DW466562 and DW435092) libraries that initiate at the LINE1 antisense promoter like LCT13 and extend past the *TFPI-2* gene with a putative alternative transcript GNGT1-005 also annotated. (**B**) Expression of TFPI-2as (upper) and *TFPI-2* (lower) in normal breast (N) and in breast cancer cell lines (H, HCC-1954; M, MCF7) analysed by strand specific and real-time RT–PCR, respectively. *TFPI-2* expression is reduced in both breast cancer cell lines compared to normal controls (n = 3). *TFPI-2* expression levels were normalized to *HPRT*. (**C**) Expression of TFPI-2as (upper) and *TFPI-2* (lower) in a panel of five matched normal and tumour breast tissue analysed as described in B.

In a previous screen for L1 chimeric transcripts, we identified a partial transcript, LCT13, present in breast and colon cancer but not in normal samples from the same tissue. LCT13 initiates at the ASP of a full-length, intergenic L1PA2 on chromosome 7 (Figure 1) (22). Annotation in the GRCh37/hg19 release of the human genome reveals an SVA_D element insertion within ORF2 rendering the L1 retrotransposition incompetent, however, maintaining an intact promoter (Supplementary Figure S1). In addition to LCT13, there are three spliced expressed sequence tags (ESTs) mapping to this locus (BG432114 from kidney; DW466562 and DW435092 from liver), which overlap with LCT13 in the 5′ region derived from the L1 element. Two of these ESTs, BG432114 and DW466562, contain exons in the *GNGT1* gene located about 300 kb telomeric to LCT13, and a putative alternative transcript spanning the locus has been annotated (GNGT1-005 ID: ENST00000455502).

Between LCT13 and *GNGT1,* in the antisense orientation, lies the tissue factor pathway inhibitor-2 gene (*TFPI-2*). The TFPI-2 protein is a protease inhibitor with a role in extracellular matrix (ECM) remodelling (40). *TFPI-2* is frequently silenced in cancer (41) and has been proposed to act as an anti-metastasis gene as its down-regulation may contribute to pathological processes such as tumour invasion and metastasis (42,43). Given that antisense transcripts have been shown to silence genes in diseases including cancer (34,35) and that EST annotations indicate the potential for large antisense transcripts in this region, we investigated whether LCT13 transcripts originating at the L1-ASP could act as antisense transcripts to *TFPI-2*. Using strand-specific RT–PCR, we observed transcription of *TFPI-2* antisense RNAs (TFPI-2as, Figure 1B, upper panel) in two breast cancer cell lines (MCF-7 and HCC-1954) previously shown to express LCT13 (22). We also determined the composite levels of *TFPI-2* sense expression compared to normal breast tissue by real-time RT–PCR (Figure 1B, lower panel). Expression of TFPI-2as correlated with decreased levels of *TFPI-2* expression in both cell lines, while normal breast, which had no detectable TFPI-2as, expressed high levels of *TFPI-2* (Figure 1B, upper versus lower panel). Similarly, analysis of total RNA from breast cancer and matched adjacent normal tissue from five individuals revealed the presence of TFPI-2as transcripts in two tumours (Figure 1C, upper panel; T1 and T3) that we have previously shown to express LCT13 (22), and in these cases, reduced *TFPI-2* expression was also observed (Figure 1C, lower panel). Downregulation of *TFPI-2* was also found in a third patient (patient 4) without any detectable LCT13 and TFPI-2as expression, a finding likely to reflect multiple mechanisms leading to loss of expression of this gene in cancer (41). The two remaining patients (patients 2 and 5) expressed low levels of *TFPI-2* RNA in the normal tissue with no further decrease in the tumour consistent with the lack of LCT13 expression. Our data show that in both cell lines and primary breast cancers, there is an association between LCT13 and TFPI-2as expression that correlates with downregulation of *TFPI-2*.

### LCT13 is a large RNA transcript including TFPI-2as

**Figure 2.**
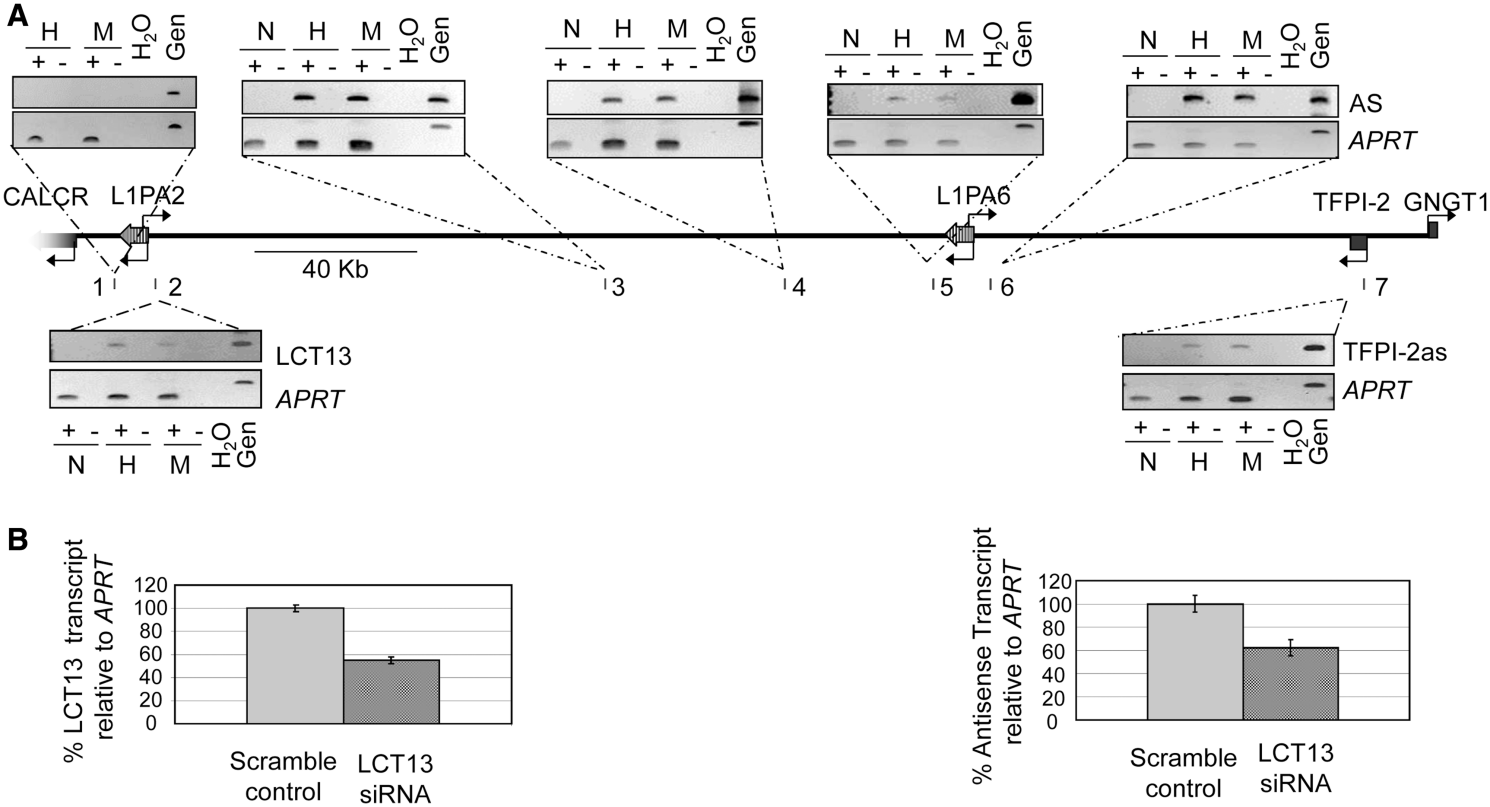
LCT13 and TFPI-2as expression is linked. (**A**) Schematic diagram of the genomic region in Figure 1A indicating regions (1–7) analysed by strand-specific RT–PCR (middle). Shown above and below the schematic are the ethidium bromide–stained gels used to visualize the strand-specific RT–PCR. Regions 2–7 are specifically expressed in cancer cell lines (H, HCC-1954 and M, MCF-7), but not normal breast (N), showing that cancer-specific antisense transcription is detectable up to 300 kb away from the *TFPI-2* gene and up to the LINE-1 retrotransposon associated with LCT13. (**B**) siRNA knockdown of the LCT13 transcript. 2D densitometry of semiquantitative strand-specific RT–PCR analysis normalized to *APRT* control reveals an approximate 50% knockdown in LCT13 levels in cells transfected with a pool of three siRNA duplexes directed against LCT13 compared to those transfected with scrambled control siRNAs (left panel). This is paralleled by a 40–50% decrease in the TFPI-2as transcript (right panel).

Given the potential for large transcripts across this locus and the restriction of LCT13 and TFPI-2as to the same cells, we hypothesized that the L1-ASP-driven LCT13 is a long transcript that extends over the entire locus including TFPI-2as. To investigate whether LCT13 and TFPI-2as are part of the same transcriptional unit, we first determined the extent of transcription in the same direction by examining seven different locations in the 300-kb region between LCT13 and TFPI-2as in normal breast and breast cancer cell lines (HCC-1954 and MCF-7) by strand-specific RT–PCR (Figure 2A). We confirmed that transcription of LCT13 and TFPI-2as (regions 2 and 7, respectively) is unique to the cancer cell lines and that no transcription in this orientation was detected centromeric to the L1PA2 associated with LCT13, consistent with our earlier finding that LCT13 transcription initiates at the L1-ASP (22). Indeed, detection of transcription both immediately upstream and downstream (regions 5 and 6) of the only other L1 element with an intact promoter in this region (L1PA6 in Figures 1A and 2B) suggests that this element is not driving the observed transcription of TFPI-2as. In all the additional regions tested (regions 3–6), we were able to detect strand-specific intergenic transcription that is restricted to cancer cells; hence, intergenic transcription centromeric to TFPI-2as extends over 300 kb up to the LINE-1 element at which LCT13 originates and is restricted to the cancer cell lines in which TFPI-2as is present. This is consistent with ENCODE RNA-seq data from MCF-7 cells showing a large RNA in this region (Supplementary Figure S2).

To further test the hypothesis that LCT13 drives transcription of TFPI-2as, we used nucleofection to introduce either a pool of three short interfering RNAs directed to the unique sequence of LCT13 or the corresponding control scrambled RNAs into MCF-7 cells. Semiquantitative RT–PCR analysis of LCT13 and TFPI-2as expression revealed that, relative to control cells, LCT13 transcript levels were decreased by approximately 50% in cells transfected with siRNAs to LCT13; this was paralleled by a 40–50% reduction in the level of TFPI-2as (Figure 2B). These findings and our observation of transcription across this locus, taken together with the annotated spliced ESTs, support the possibility that LCT13 and TFPI-2as belong to the same large RNA transcript.

**Figure 3.**
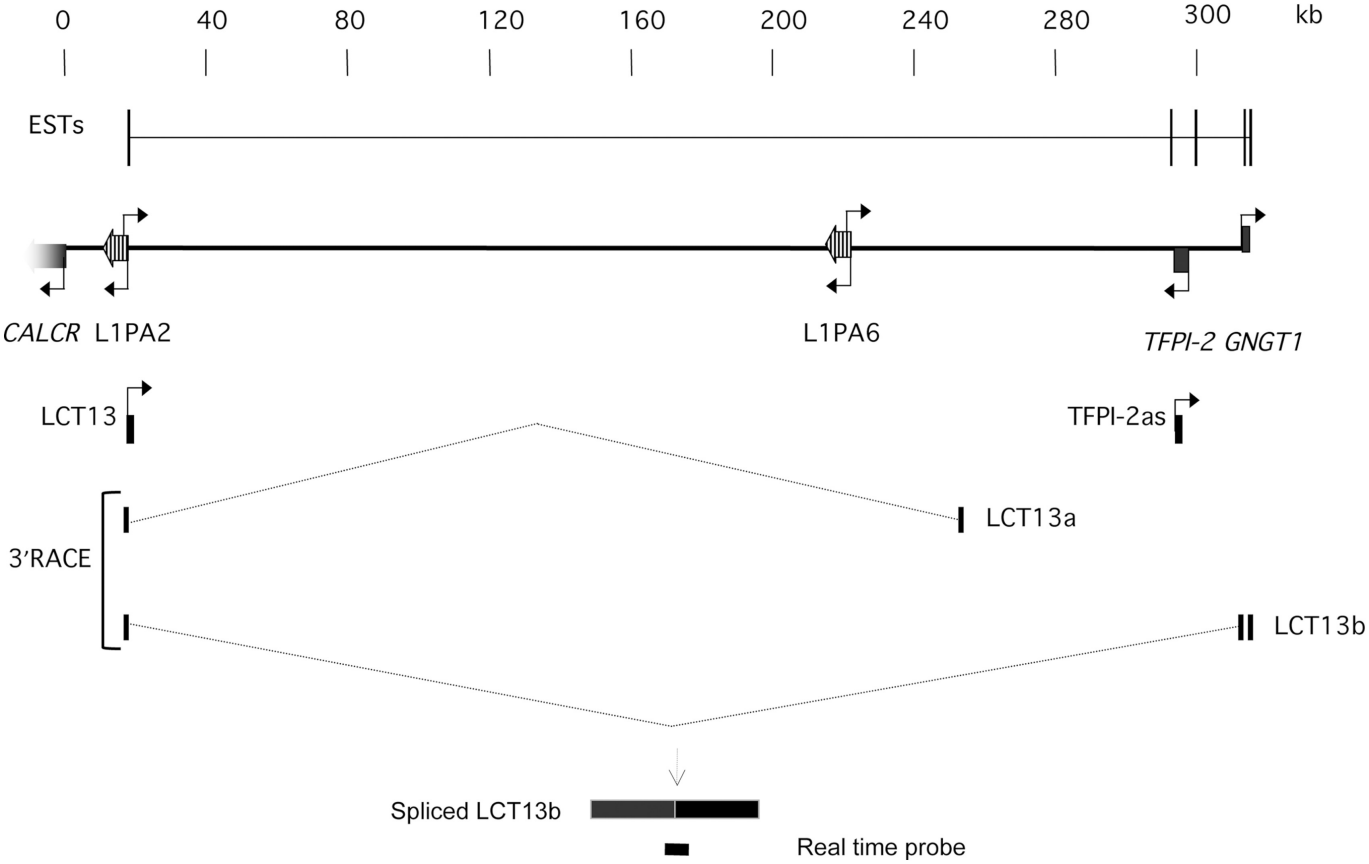
LCT13 and TFPI-2as are part of the same large transcript. Schematic diagram of the 7q21.3 region described in Figure 1A with annotated LCT13 splicing isoforms LCT13a and LCT13b isolated specifically from cancer cells by 3′ RACE anchored on LCT13. The combined ESTs described in Figure 1A are shown above for comparison purposes. Note that the LCT13b isoform includes exons 2 and 3 of the *GNGT1* gene, encompassing *TFPI-2*, consistent with the presence of uninterrupted transcription across this region in cancer cell lines. Below this is a drawing of the custom Taqman assay designed to study LCT13b expression. The probe spans the splice site between the L1PA2 LINE-1ASP and GNGT1 exon 2. Spliced LCT13b and real time probe are not drawn to scale.

Alternatively it is also feasible that LCT13 and TFPI-2as are separate transcripts and transcription of LCT13 promotes that of TFPI-2as by opening up the region as documented during recombination at immunoglobulin genes (44). To gain more conclusive evidence, we performed 3′ RACE on oligo-dT primed cDNA from normal breast, MCF-7 and HCC-1954 cells. We identified two spliced transcripts (hereafter referred to as LCT13a and LCT13b) specific to cancer cells (Figure 3B). These are partial spliced transcripts arising from dT priming within polyA tracts present in the genome, and both display coding potential (Supplementary Figures S3 and S4). We found that LCT13b contains splicing from the L1ASP to exon2 and 3 of the *GNGT1* gene, thus encompassing *TFPI-2* in the antisense orientation, confirming that LCT13 and TFPI-2as are part of the same transcriptional unit. Therefore, this cancer cell–specific transcript initiates at an L1-ASP and extends uninterrupted over more than 300 kb across the genomic locus to generate a large RNA antisense to *TFPI-2*.

### Antisense RNA can establish silencing at TFPI-2 in mouse embryonic stem cells

**Figure 4.**
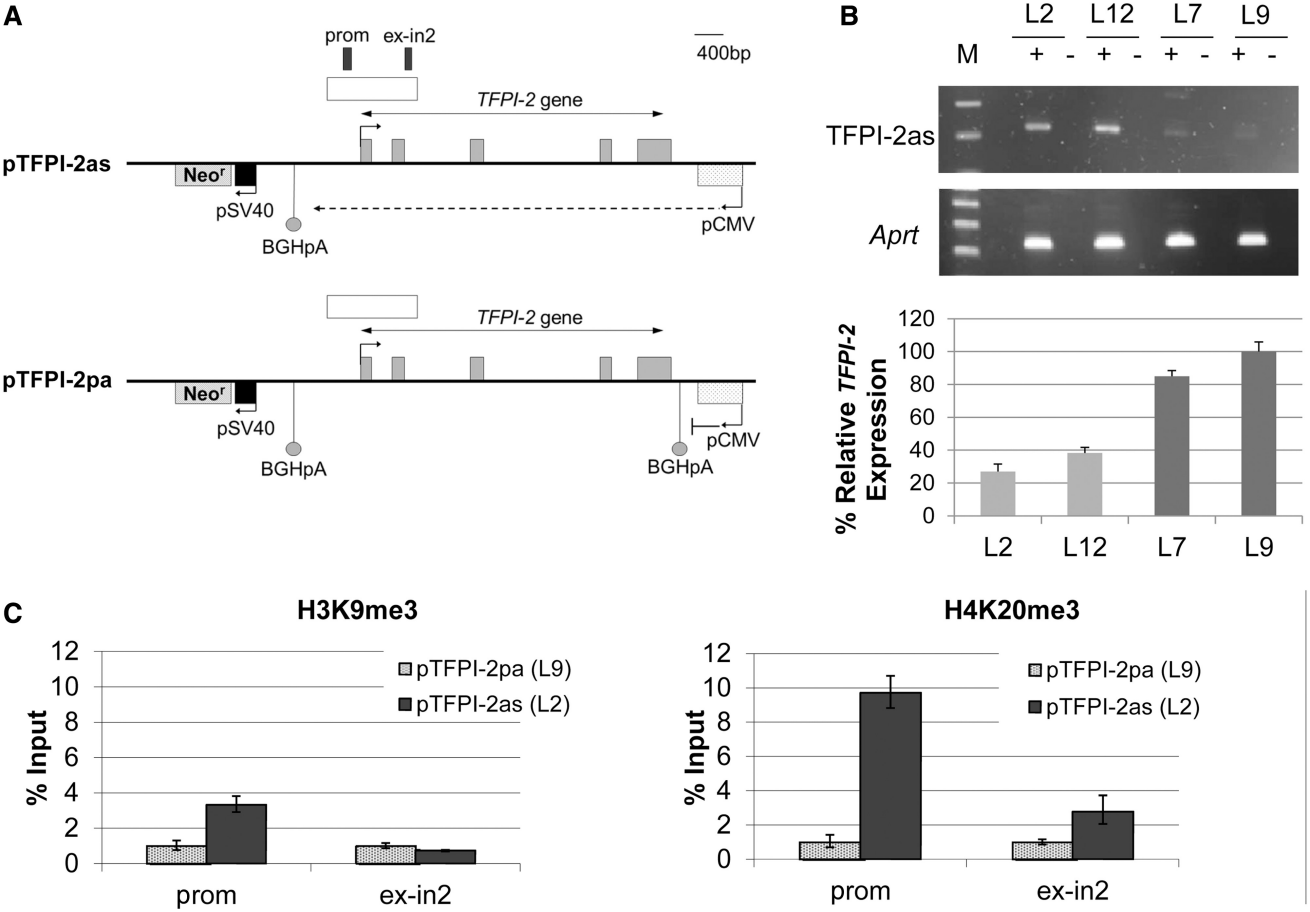
A human *TFPI-2* transgene is sensitive to antisense RNA repression in mouse ES cells. **(A)** Schematic diagram of constructs introduced into mouse ES cells: pTFPI-2as is designed to transcribe antisense to *TFPI-2* from a CMV promoter, while pTFPI-2pa has a poly-A signal insertion downstream of the CMV promoter to block antisense transcription. Arrows indicate direction of transcription. Regions analysed by ChIP are annotated as ‘prom’ and ‘ex-in2’. (**B**) Strand-specific RT–PCR analysis of TFPI-2 antisenese (TFPI-2as) expression in transgenic mouse ES cell lines demonstrates increased levels in pTFPI-2as lines (L2 and L12) relative to pTFPI-2pa cells (L7 and L9), mouse *Aprt* acts as a positive control for RNA quality and quantity. This correlates with a reduction in *TFPI-2* expression as shown by real-time PCR normalized to mouse *Gapdh*. (**C**) ChIP analysis followed by real-time PCR. Left panel: Antibodies to H3K9me3 reveal localized enrichment of H3K9me3 in the promoter region in the antisense expressing cell line, pTFPI-2as (L2), compared to cells transfected with pTFPI-2pa (L9), which express low levels of TFPI-2as. Right panel: Antibodies to H4K20me3 also show enrichment at the *TFPI-2* promoter in pTFPI-2as compared to pTFPI-2pa.

We wanted to investigate whether antisense RNAs can lead to the establishment of silencing at *TFPI-2*. To this aim, we utilized a transgenic mouse ES cell system, a well-established model for the investigation of antisense RNA and transcription in epigenetic gene silencing (34,35,45). We generated lines stably transfected with one of two constructs: pTFPI-2as, containing the full-length *TFPI-2* gene, including promoter and poly-A signals (46), with antisense transcription driven by the cytomegalovirus promoter or pTFPI-2pa, a control construct, identical to pTFPI-2as except for the introduction of the BGH poly-A signal to prevent antisense transcription through the *TFPI-2* gene (Figure 4A). The CMV promoter was chosen because it allows stable expression of transgenes in mouse ES cells (47) and has been used in these cells to study the functional role of antisense RNA at the *p15* gene in cancer (35). To favour integration of the construct within permissive chromatin, the neomycin resistance gene driven by the SV40 promoter was included in the fragment used for electroporation (Figure 4A), and selection was maintained throughout the experiment.

We found that in cell lines expressing TFPI-2as RNA (L2 and L12), *TFPI-2* was repressed, while cell lines with the pTFPI-2pa construct (L7 and L9) and relatively low levels of antisense transcript expressed higher levels of *TFPI-2*, confirming that antisense RNA can directly induce *TFPI-2* gene silencing in this model (Figure 4B). These results are consistent with previous observations that randomly integrated constructs with convergent transcription reproducibly display transcriptional silencing (34,35).

It has been proposed that antisense RNA can drive gene silencing by triggering the deposition of repressive histone marks (35,48); therefore, we looked for differences in histone modifications in ES cells with and without the antisense RNA. We observed that decreased *TFPI-2* expression corresponds to a relative increase in H3K9me3 and a marked increase in H4K20me3 within the *TFPI-2* CGI in mouse ES cells expressing the antisense RNA (pTFPI-2as line L2) compared to control cells with low levels of antisense RNA (pTFPI-2pa line L9) (Figure 4C). Similar to previous observations (35), the changes in histone modification seem to be the primary consequence of antisense RNA-mediated silencing in this model system, with DNA methylation being induced at the silenced locus on differentiation. Indeed, using bisulphite sequencing, we could not detect any changes in DNA methylation at the *TFPI-2* promoter in ES cells, which were virtually devoid of methylation, but DNA methylation was only evident in day 7 embryoid bodies derived from the pTFPI-2as lines (Supplementary Figure S5). Therefore, in this model system, expression of an antisense RNA to *TFPI-2* can induce gene repression via deposition of repressive histone marks, indicating that the *TFPI-2* promoter is susceptible to this form of silencing.

### LCT13 expression is linked to silencing of TFPI-2 in breast cancer cell lines

We wanted to explore whether cancer cell lines expressing LCT13 have changes in histone modifications paralleling those in the ES cell model. First, to examine *TFPI-2* antisense transcription in a more quantitative fashion, we designed a real-time assay probe spanning the exon1–exon2 boundary of LCT13b (real-time probe in Figure 3B). As *TFPI-2* lies within intron 1 of LCT13b (Figure 3A), we reasoned that analysis of expression of LCT13b could act as a good proxy for the detection of antisense transcription through *TFPI-2*. We validated this assay on random primed RNA from breast cancer cell lines and normal human mammary epithelial cells (HMECs) (Supplementary Figure S6) confirming our earlier observations (Figure 1B) that increased antisense expression in breast cancer cell lines MCF-7 and HCC-1954 is associated with decreased *TFPI-2* expression, proving its validity. Moreover we were able to extend these findings to an additional breast cancer cell line, T47D, and normal HMEC cells. We found that T47D expresses LCT13 at very high levels but no detectable *TFPI-2* expression while LCT13 is not present in HMEC, which express higher levels of *TFPI-2* (Supplementary Figure S6).

**Figure 5.**
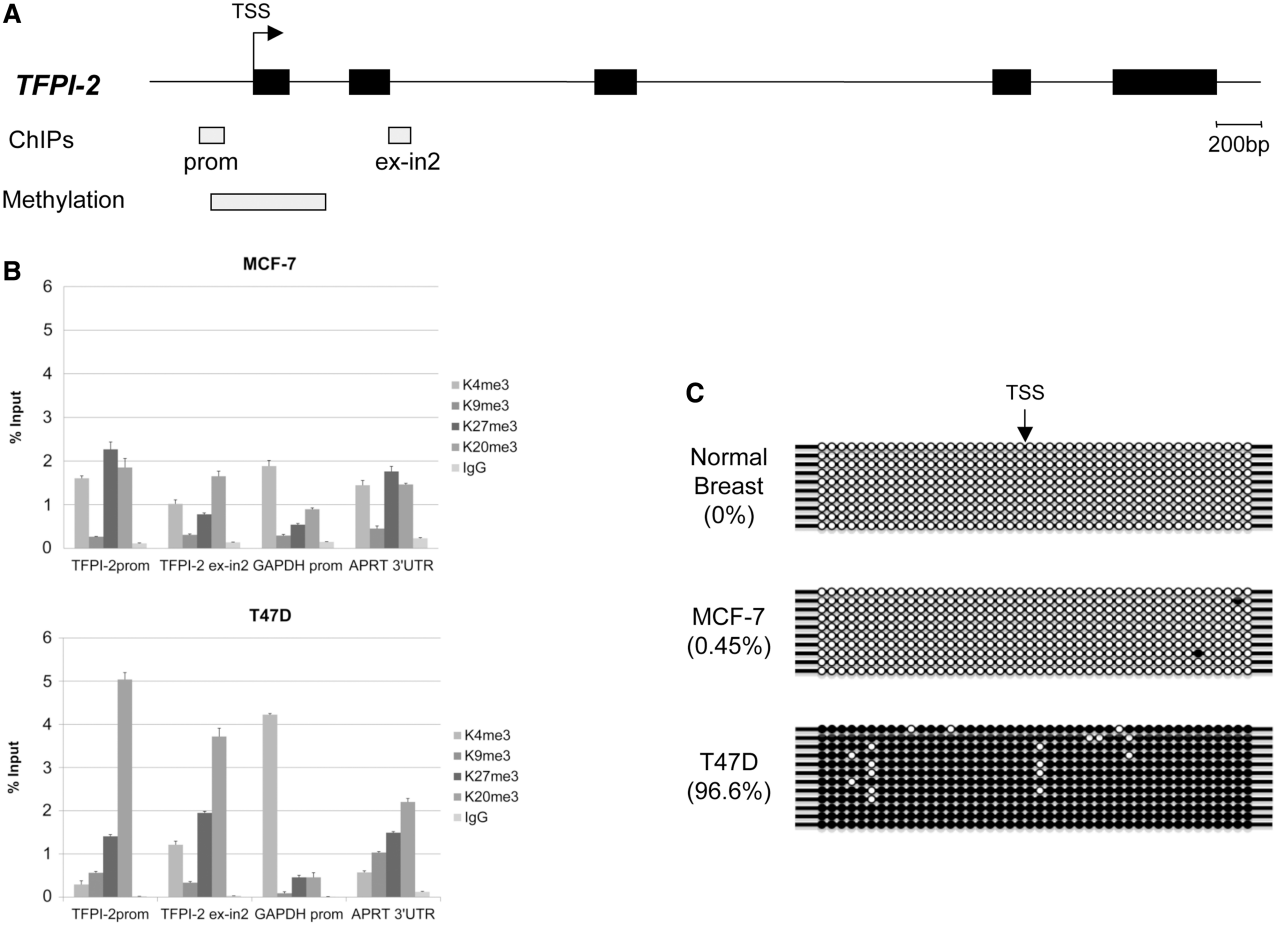
Silencing of *TFPI-2* in breast cancer. (**A**) Top panel: Schematic diagram of the *TFPI-2* gene. Black boxes on the line represent exons of *TFPI-2*, and the bent arrow indicates the TSS. Light grey boxes labelled ChIP and methylation denote regions of the *TFPI-2* CpG island analysed by ChIP and bisulphite sequencing, respectively. **(B)** ChIP assays in MCF-7 (top) and T47D (bottom) cells using antibodies against repressive histone marks H3K9me3, H3K27me3, H4K20me3, and active marks AcH3 and H3K4me3. Enrichment of H3K27me3 and H4K20me3 is seen in both cell lines at the *TFPI-2* regions tested. (**C**) Bisulphite sequencing of DNA from normal breast, MCF-7 and T47D cells, shows that the *TFPI-2* region studied is devoid of methylation in normal breast (0%) and MCF-7 cells (0.4%) but heavily methylated in T47D cells (96%). TSS indicates the transcriptional start site of the *TFPI-2* gene; filled and open circles represent methylated and unmethylated CpG dinucleotides, respectively. Each row represents an independent clone.

We examined two regions of the *TFPI-2* gene CGI in the promoter (prom in Figure 5A) and spanning the exon–intron boundary of exon2 (ex-in2 in Figure 5A) by chromatin immunoprecipitation in LCT13 expressing MCF-7 and T47D cells. Antibodies against the active histone modification H3K4me3 and the repressive histone modifications H3K9me3, H3K27me3 and H4K20me3 were tested. Enrichment in H3K27me3 and H4K20me3 was observed at both positions in MCF7 cells (Figure 5B, top panel). A more marked increase in H4K20me3 and comparable increase in H3K27me3 were observed in T47D cells at both positions (Figure 5B, bottom panel) suggesting that this region is associated with heterochromatic marks in both cell lines. This is consistent with data available from the ENCODE project that also shows differences in the levels of the heterochromatic mark H3K27me3 between normal cells (HMEC), which are devoid of this mark and MCF-7 cancer cells that display H3K27me3 enrichment at *TFPI-2* (Supplementary Figure S7).

We examined the methylation status of a segment of the *TFPI-2* CGI spanning the proximal promoter, the transcription start site (TSS), exon 1 and part of intron 1 (Methylation in Figure 5A), in normal breast tissue, MCF-7 and T47D breast cancer cells. Bisulphite sequencing reveals that methylation levels in MCF-7 breast cancer cells were negligible and comparable to those of normal breast (0.47 and 0% methylation, respectively; Figure 5C). In contrast, high levels of DNA methylation (96.6%) were observed in the T47D cell line (Figure 5C), indicating that repression of *TFPI-2* mediated by expression of the long LCT13 RNA in breast cancer cells can in some instances lead to subsequent methylation of the *TFPI-2* CGI, which may reflect dissimilarities in differentiation potential between the MCF-7 and T47D cell lines. Consistent with this, the presence of significant H3K4me3 and H3K27me3 at the *TFPI-2* promoter in MCF-7 cells suggests that repression of *TFPI-2* is marked by bivalent histone modifications similar to those observed in pluripotent cells. Indeed, this is confirmed by analysis of available ENCODE datasets revealing bivalency at the *TFPI-2* promoter in MCF-7 cells (Supplementary Data, Figure S7). This bivalency is resolved in T47D cells, where H3K4me3 is reduced but there is now DNA methylation (as observed in differentiated ES cells), which is incompatible with H3K4me3 (49).

Taken together our data suggest that LCT13 may induce *TFPI-2* repression by triggering changes in chromatin conformation via deposition of epigenetic modifications in MCF-7 and T47D cells.

### LCT13 is not required for maintenance of TFPI-2 silencing in MCF-7 cells

**Figure 6.**
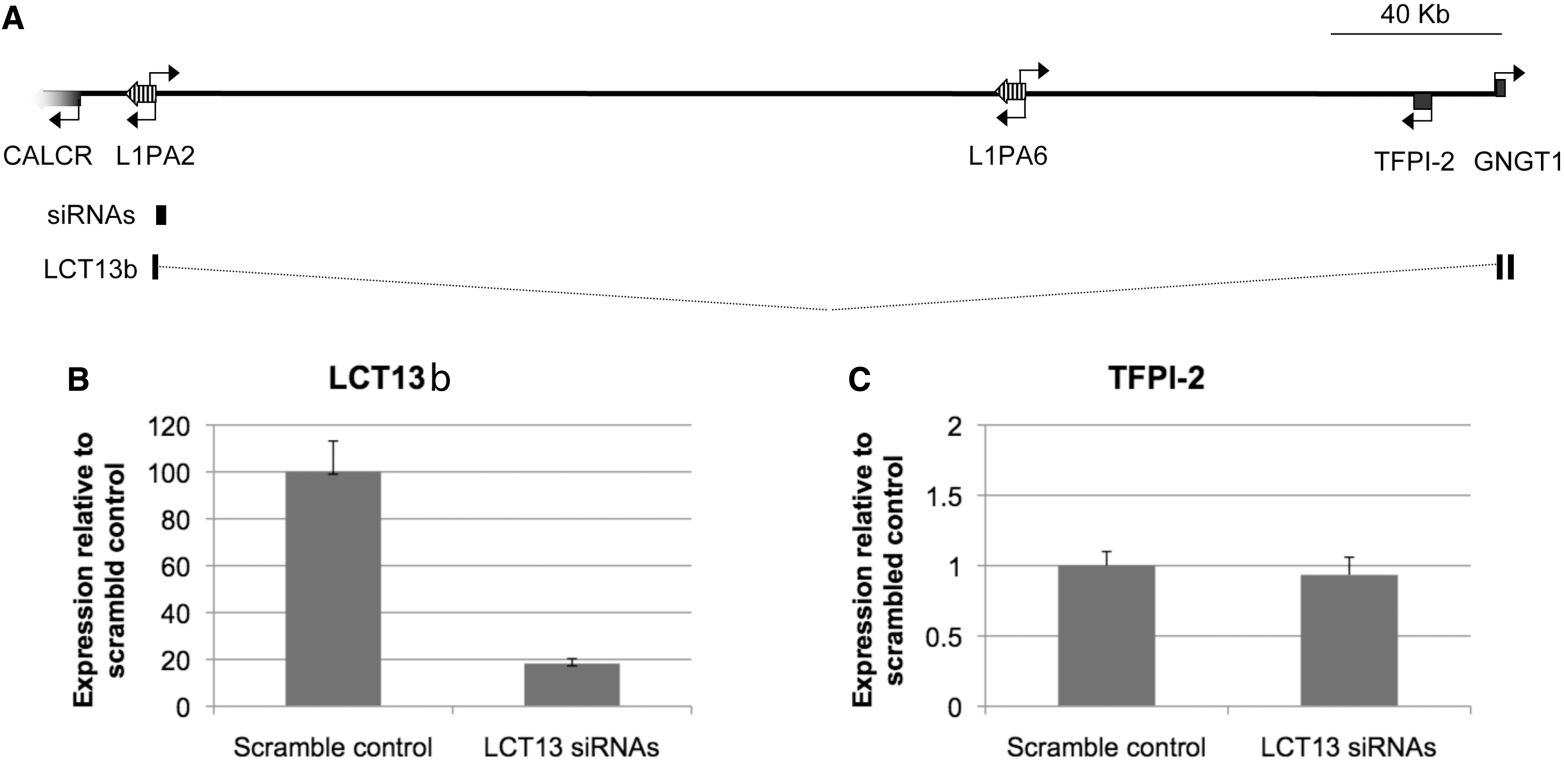
LCT13 is not required for maintenance of silencing at *TFPI-2*. (**A**) Schematic diagram of the 7q21.3 region described in Figure 1A drawn to scale (bar is 40 kb) with the LCT13b transcript and the site targeted by the three independent siRNAs annotated. (**B**) Expression of LCT13 and (**C**) expression of *TFPI-2* in MCF-7 cells treated with a pool of three LCT13 siRNAs or the equivalent scrambled control show that decreased LCT13b levels do not correspond to an increase in *TFPI-2* in these cells.

We next wanted to gain further experimental insights into the involvement of LCT13 in repression of *TFPI-2*. To investigate whether expression of the large antisense transcript is required to maintain *TFPI-2* silencing in MCF-7 cells, we analysed LCT13b and *TFPI-*2 expression by real-time RT–PCR in MCF-7 cells treated with a pool of three short interfering RNAs directed to the unique sequence of LCT13 as described above (Figure 2B and siRNAs in Figure 6A). Treatment with siRNAs resulted in a 5.5-fold reduction in LCT13b expression relative to control cells treated with siRNAs to the equivalent scrambled sequence (Figure 6B); however, no significant increase in *TFPI-2* expression was observed in cells treated with the LCT13 siRNA pools relative to scrambled control treated cells (Figure 6C). These results suggest that LCT13 antisense RNA is not required to maintain silencing of *TFPI-2* in MCF-7 cells; a finding similar to antisense-mediated repression at p15 (35).

Given the potential for the formation of double-stranded RNAs at this locus, we looked for the presence of small RNAs in LCT13 expressing breast cancer cells by northern blot analysis (Supplementary Data, Figure S8). Despite readily detecting a control small RNA, we were unable to identify any small RNAs derived from this region. Given that convergent transcription has been shown to induce transcriptional gene silencing in a Dicer-dependent manner via production of double-stranded RNAs (50), it is possible that siRNAs may play a role in the establishment but not the maintenance of silencing, consistent with our previous finding that maintenance of silencing at *TFPI-2* is independent of the antisense-RNA (Figure 6C). Alternatively these data could point to a Dicer-independent mechanism of antisense-RNA–mediated repression as suggested at the p15 locus (35) or indicate that antisense transcription rather than the antisense RNA is required for the establishment of silencing, similar to observations at the Igf2R locus (51). Future experiments will determine what is responsible for *TFPI-2* silencing in this system, whether antisense transcription or the antisense-RNA and, if the latter, whether Dicer is involved.

### LCT13 expression is linked to silencing of TFPI-2 in colorectal cancer

**Figure 7.**
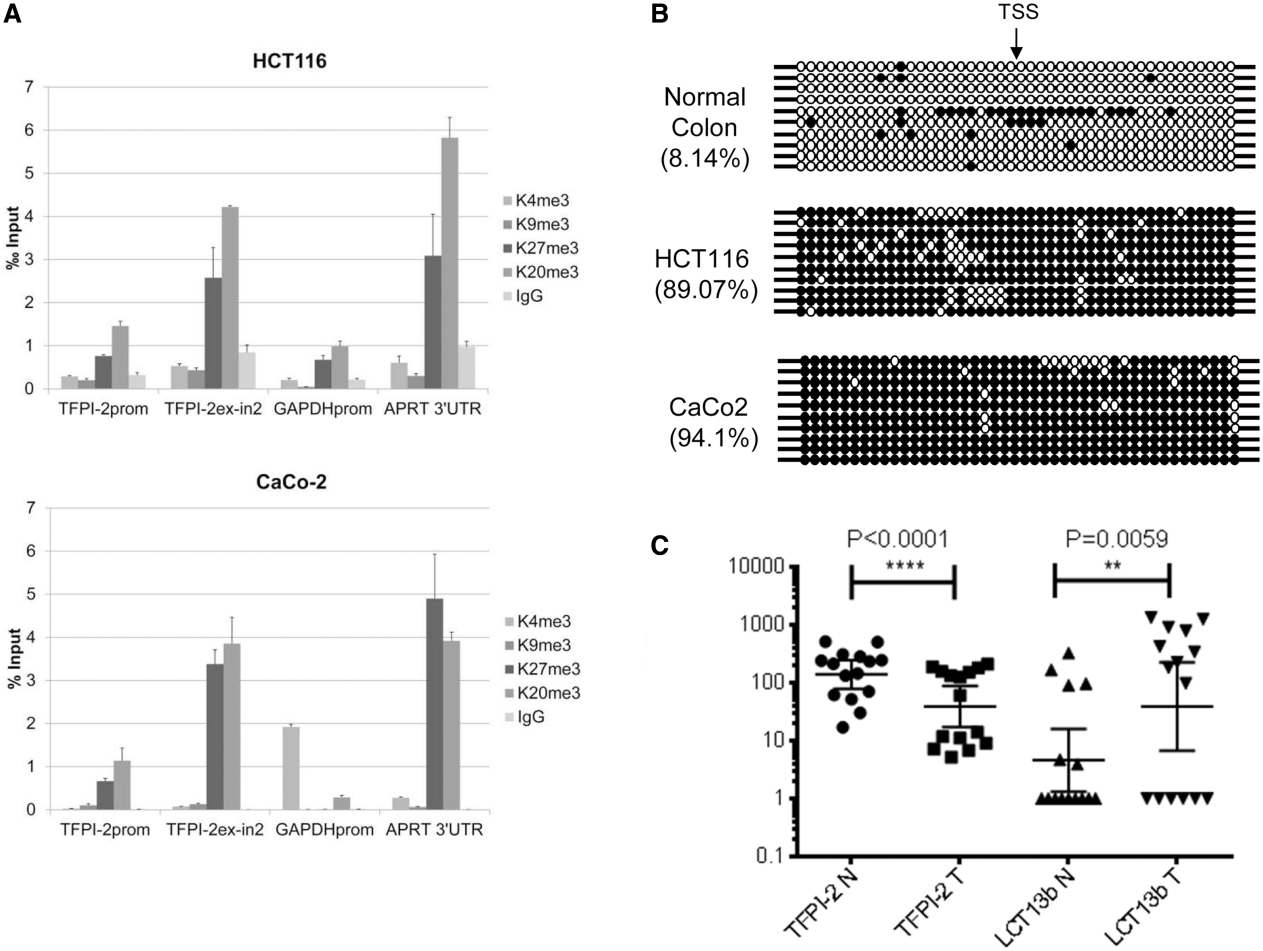
Silencing of *TFPI-2* in colorectal cancer. (**A**) Chromatin immunoprecipitation assays in HCT116 (top) and CaCo-2 (bottom) cells using antibodies against active histone marks AcH3, H3K4me3, and repressive histone marks H3K9me3, H3K27me3, and H4K20me3 indicate enrichment of repressive marks H3K27me3 and H4K20me3 at the *TFPI-2* regions tested (see Figure 5A). (**B**) Bisulphite sequencing analysis of 10 independent clones from normal colon and HCT116 and CaCo-2 colon cancer cells shows that the *TFPI-2* region studied is hypomethylated in normal colon (8.14% methylation) and hypermethylated in HCT116 and CaCo-2 cells (89.07 and 94.1% methylation, respectively). TSS indicates the transcriptional start site of the *TFPI-2* gene; filled and open circles represent methylated and unmethylated CpG dinucleotides, respectively. Each row represents an independent clone. The region analysed by bisulphite sequencing is depicted in Figure 5A. (**C**) Analysis of LCT13 and *TFPI-2* expression in matched normal and tumour tissues from sporadic cases of colorectal cancer shows an inverse relationship with a significant increase in LCT13 (*P* < 0.007, Wilcoxon test, *n* = 15) corresponding to a significant decrease in *TFPI-2* in tumours. Real-time RT–PCR expression data was normalized to *PGK1*, *GAPDH*, and *HPRT* using GenEx software (Multid Analyses AB).

We have previously shown that expression of LCT13 is not restricted to breast cancer but is also detected in colon cancer cell lines (22). By using the LCT13b Taqman assay, we analysed HCT116 and CaCo-2 colon cancer cells and found that expression of LCT13b is also associated with down-regulation of *TFPI-2* in these lines (Supplementary Data, Figure S9). In LCT13b expressing HCT116 and CaCo-2 cells, the *TFPI-2* promoter is enriched in repressive histone modifications (H3K27me3 and H4K20me3) (Figure 7A). For both HCT116 and CaCo-2 cells, there was no significant enrichment in H3K4me3 at the *TFPI-2* promoter. Indeed, ChIP data available from ENCODE for these colon cancer cells is consistent with our findings (Supplementary Figure S7). Due to restrictions in our ethical approval, we are unable to perform ChIP experiments on normal colon tissue; however, ChIP data on normal colon mucosa available from the Roadmap Epigenomics Project reveals no significant enrichment in repressive modifications at the *TFPI-2* promoter in normal colon (Supplementary Figure S10). Furthermore, like T47D, the colon cancer cell lines tested also lack H3K4me3 and are heavily methylated (Figure 7B). Taken together, these observations suggest that antisense-driven silencing of *TFPI-2* is not restricted to breast cancer and may also occur in colon cancer.

To investigate the *in vivo* relevance of such findings, we analysed LCT13b and *TFPI-2* expression levels by real-time RT–PCR in matched normal and tumour colorectal tissue samples from 27 sporadic cases of colorectal cancer. Fifteen of twenty-seven patients (∼56%) displayed significant downregulation of *TFPI-2* in tumours (*P* < 0.0001, Wilcoxon matched-pairs signed rank test). Of these patients with downregulated *TFPI-2*, there is an association with a significant increase in LCT13b RNA levels (*P* = 0.0059, Wilcoxon matched-pairs signed rank test) (Figure 7C). This finding is consistent with our earlier observation in a smaller cohort of breast cancer samples (Figure 1C) and indicates that in a significant proportion of the colorectal cancer cases with *TFPI-2* silencing, there is an association with expression of the antisense RNA LCT13.

## DISCUSSION

TFPI-2 is a Kunitz-type serine protease inhibitor homologous to tissue factor pathway inhibitor (TFPI) (52,53), and its down-regulation in cancer has been proposed to contribute to tumour malignancy due its role in ECM remodelling. This is supported by the observation that decreased levels of *TFPI-2* associate with increased tumour invasion and metastasis in a number of cancers, particularly more aggressive cancers such as glioma (42) and pancreatic cancer (42,43). Consistently, ectopic expression of *TFPI-2* can inhibit tumour growth and metastasis *in vivo* by regulating pericellular ECM remodelling and angiogenesis (54).

Different molecular mechanisms can lead to loss of *TFPI-2* expression in cancer, including deletions, mutations in the promoter (41) and epigenetic silencing (55), and recent evidence indicates that *TFPI-2* can also be downregulated by miR-616 in prostate cancer (56). In this study, we propose an additional mechanism for *TFPI-2* repression in cancer: expression of an aberrant transcript, LCT13, initiating at the ASP of an intergenic LINE-1 element and extending over 300 kb, antisense to the *TFPI-2* gene, is associated with *TFPI-2* repression and the deposition of repressive histone marks. Indeed recent evidence suggests a role for antisense RNA in the targeting of polycomb repressor complexes (57). Future work will determine whether such complexes are involved this case. Interestingly, depending on the cell type, antisense expression was associated with either a methylated or unmethylated CGI indicating that methylation of the CGI is not essential for repression. This result is consistent with previous findings that *TFPI-2* silencing can occur without changes in DNA methylation within the proximal promoter region (55). For example, compared to matched normal liver, human hepatocellular carcinomas (HCC) significantly underexpress *TFPI-2* in approximately 90% of cases; however, more than 50% of HCCs retain a non-methylated promoter (58). Our DNA methylation findings are in line with the fact that decreased LINE-1 methylation does not appear to correlate with hypermethylation of gene promoter CGIs (59) and may signify that DNA methylation is an indirect consequence of the epigenetic silencing brought about by the repressive histone modifications; supported by our ES cell data and published work on antisense RNA-mediated silencing of p15 in cancer (35). The heterogeneous patterns of DNA methylation may reflect cellular origin as has been previously observed in cancer (6,7), and their functional impact is a subject of intense interest and speculation.

Given the repetitive nature of LINE-1 elements within the genome, this mechanism may be more widespread and not restricted to *TFPI-2*. Antisense RNAs have been previously observed to silence genes (34,35) and transposable elements are the source of the majority of non-coding antisense transcripts (26). In this context, it is tempting to speculate that LINE-1 driven transcripts are implicated in some instances of long-range silencing reported in cancer (60), pointing to a novel retrotransposition independent role for LINE-1 activation in cancer. An intragenic L1-ASP has been shown to act as an alternate promoter in cancer driving the transcription of a truncated and oncogenic isoform of the *c-MET* gene (28,30,61), and the ability of retrotransposons to act as alternative promoters for protein coding genes has been reported (20). Indeed, the LCT13 L1ASP could potentially act as an alternative promoter for *GNGT1* (Supplementary Data, Figures S3 and S4), but further work is necessary to confirm whether LCT13b is polyadenylated and translated. Nevertheless our data show that LCT13 acts as an antisense RNA linked to *TFPI-2* downregulation indicating that L1-derived transcripts can affect gene expression patterns in cancer by multiple mechanisms.

In conclusion, activation of a LINE-1 element and silencing of a tumour suppressor gene may be causally linked events in cancer, suggesting a widespread mechanism, whereby aberrantly active LINE-1 promoters drive RNA transcription over long distances and trigger epigenetic silencing of protective genes; thus promoting cancer progression. What triggers activation of LINE-1 promoters in cancer remains a fundamental and unanswered question. It is widely believed that LINE-1 activation is a consequence of genome-wide hypomethylation observed in cancer, and indeed methylation has been proposed as a defence mechanism against transposable elements (62). However, the mechanisms leading to genome-wide hypomethylation are unclear and also whether the same mechanisms are responsible for activation of transposition proficient and deficient LINE-1 elements is still not known. Further studies exploiting LCTs like LCT13 will make addressing these questions possible by studying individual LINE-1 promoters. Finally, it is worth noting that as the majority of non-coding RNAs initiate at retrotransposable elements (26), LINE-1 retrotransposon-driven transcription may play important regulatory roles in normal development and differentiation at stages and in tissues where LINE-1 elements are active (e.g. germ cells, embryonic stem cells and pre-implantation stages) (63). Therefore, the LINE-1-driven epigenetic effects described in our article maybe more widespread than previously thought and as relevant to normal development and differentiation as to disease progression.

## SUPPLEMENTARY DATA

Supplementary Data are available at NAR Online: Supplementary Tables 1–5, Supplementary Figures 1–10, Supplementary Method and Supplementary References [64,65].

## FUNDING

Cancer Research UK Project Grant and a Royal Society Dorothy Hodgkin Research Fellowship (C.T.); Research in RM’s lab is supported by the MRC and Breakthrough Breast Cancer. Funding for open access charge: University of Nottingham.

*Conflict of interest statement.* None declared.

## Supplementary Material

Supplementary Data
