# Supplementary material for: Expression of a large LINE-1-driven antisense RNA is linked to epigenetic silencing of the metastasis suppressor gene TFPI-2 in cancer
Source: Nucleic Acids Res. 2013 May 23;41(14):6857–69. doi: 10.1093/nar/gkt438 (PMC3737543; doi:10.1093/nar/gkt438)
Supplement: Supplementary Data [file supp_41_14_6857__index.html]

Expression of a large LINE-1-driven antisense RNA is linked to epigenetic silencing of the metastasis suppressor gene TFPI-2 in cancer — Expression of a large LINE-1-driven antisense RNA is linked to epigenetic silencing of the metastasis suppressor gene TFPI-2 in cancer — Supplementary Data 

# Expression of a large LINE-1-driven antisense RNA is linked to epigenetic silencing of the metastasis suppressor gene *TFPI-2* in cancer

## Supplementary Data

files

**Files in this Data Supplement:**

- Supplementary Data - pdf file
