# Supplementary material for: Expression of a large LINE-1-driven antisense RNA is linked to epigenetic silencing of the metastasis suppressor gene TFPI-2 in cancer
Source: Nucleic Acids Res. 2013 May 23;41(14):6857–69. doi: 10.1093/nar/gkt438 (PMC3737543; doi:10.1093/nar/gkt438)
Supplement: Supplementary Data [file supp_gkt438_nar-00130-x-2013-File009.pdf]

Supplementary Data

Table of Contents

Supplementary Method 2

Northern Blotting for Small RNAs........ 2

References 2

Supplementary Figures........ 3

Supplementary Figure 1: Structure of the L1PA2 associated with LCT13 3

Supplementary Figure 2: Evidence of transcription across the GNGT1/TFPI-2 locus from ENCODE 4

Supplementary Figure 3: The LCT13a transcript 5

Supplementary Figure 4: The LCT13b transcript 6

Supplementary Figure 5: Methylation of TFPI-2 in differentiating pTFPI-2as and pTFPI-2pa ES cells........ 7

Supplementary Figure 6: Expression of LCT13b and TFPI-2 in breast cancer cell lines 8

Supplementary Figure 7: ChIP data at the TFPI-2 locus from ENCODE........ 9

Supplementary Figure 8: Lack of detection of TFPI-2 small RNAs in breast cancer cell lines........ 10

Supplementary Figure 9: Expression of LCT13b and TFPI-2 in colon cancer cell lines.... 11

Supplementary Figure 10: ChIP data for normal colon mucosa generated by the Roadmap Epigenomics Project........ 12

Supplementary Tables........ 13

Supplementary Table 1. List of primers used in strand specific reverse transcription........ 13

Supplementary Table 2. PCR primer pairs 14

Supplementary Table 3. Primers used for amplification of bisulphite treated DNA 15

Supplementary Table 4. Primers used with Roche multiprobe for real time PCR following ChIP 15

Supplementary Table 5. Taqman assays used in the study........ 15

Supplementary Method

Northern Blotting for Small RNAs

Low molecular weight RNAs were size fractionated from total RNA using published protocols (64). 20µg samples of low molecular weight RNA were separated by electrophoresis on 15% denaturing polyacrylamide (19:1) gels with 7mM urea and buffered with 20mM MOPS/NaOH (pH 7). RNA was transferred and crosslinked to nylon membranes as published (65). Membranes were hybridised with oligonulceotide probes labeled with ^{32}P using T4 polynucleotide kinase (NEB) in 5 x SSPE, 10 x Denhardt's solution, 0.1% SDS and 40µg/ml salmon sperm DNA at an appropriate temperature overnight. Membranes were washed in 5 x SSPE twice at room temperature and exposed to autoradiograph film (Kodak) for imaging.

References

64. Lu, C., Meyers, B.C. and Green, P.J. (2007) Construction of small RNA cDNA libraries for deep sequencing. *Methods*, **43**, 110-117.

65. Pall, G.S., Codony-Servat, C., Byrne, J., Ritchie, L. and Hamilton, A. (2007) Carbodiimide-mediated cross-linking of RNA to nylon membranes improves the detection of siRNA, miRNA and piRNA by northern blot. *Nucleic Acids Res*, **35**, e60.

Supplementary Figures

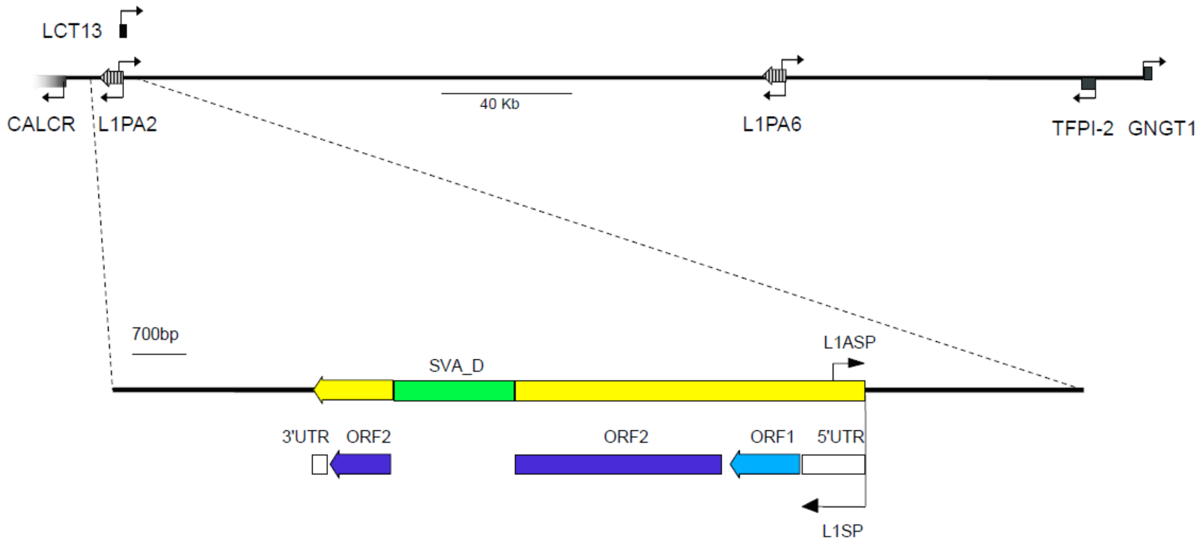

Supplementary Figure 1: Structure of the L1PA2 associated with LCT13. At the top is the schematic diagram of a 300kb region of chromosome 7q21.3 including LCT13 and the *TFPI-2* gene with scale indicated. Below this is the enlargement of the region including L1PA2 (yellow) showing its structure with the SVA_D (green) insertion within ORF2 (dark blue box and block arrow). Bent arrows indicate the L1 sense (L1SP) and antisense (L1ASP) promoters, 5' and 3' UTRs are shown as white boxes and ORF1 as light blue block arrow.

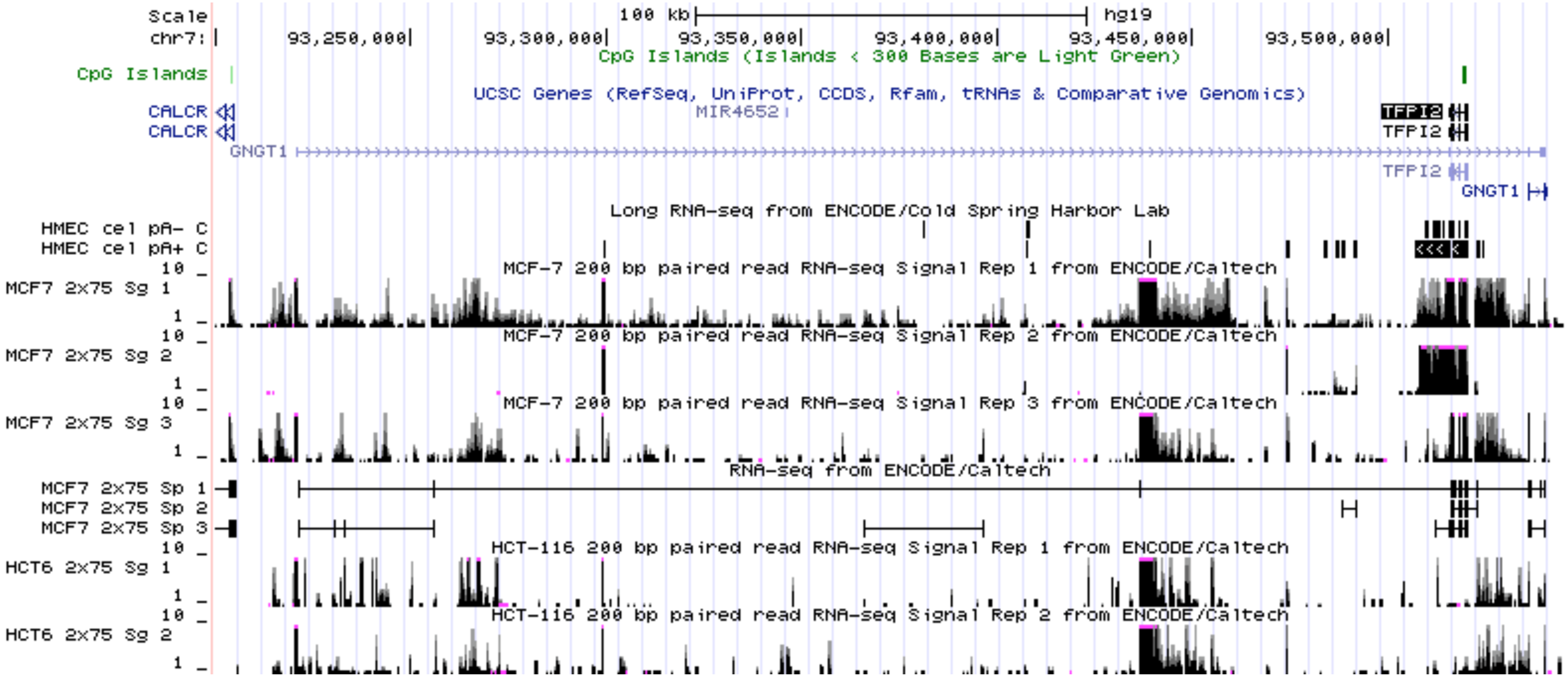

Supplementary Figure 2: Evidence of transcription across the GNGT1/TFPI-2 locus from ENCODE. UCSC Genome browser snapshot of the GNGT1/*TFPI-2* locus displaying ENCODE RNAseq traces for HMEC, MCF-7, and HCT116 (HCT6 traces) cells. HMEC traces show the presence of annotated reads predominantly in the *TFPI-2* gene. In contrast, RNAseq traces for MCF-7 and HCT116 cells have reads annotated throughout the locus. This is consistent with the presence of a long RNA transcript in the GNGT1/*TFPI-2* region in MCF-7 and HCT116 cells but not in HMEC cells, similar to our findings (Supplementary Fig 6).

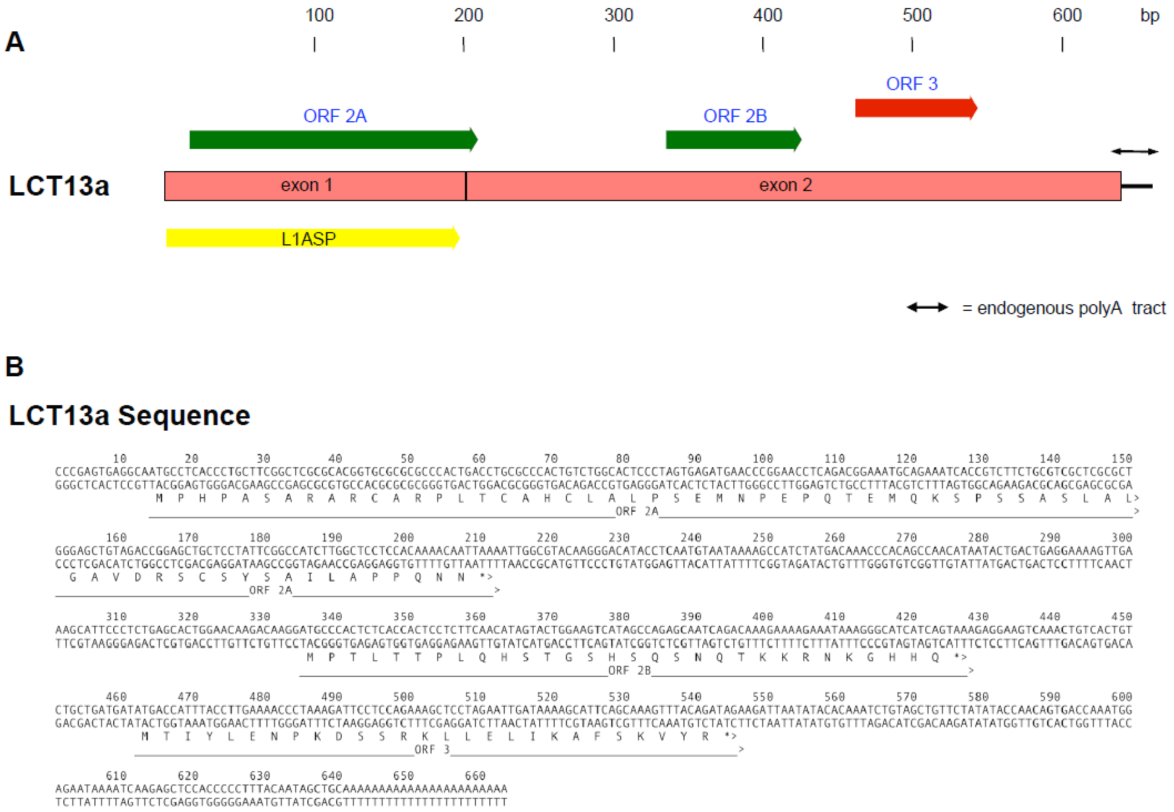

Supplementary Figure 3: The LCT13a transcript. **(A)** Schematic diagram depicting the exon structure of the LCT13a transcript with indicated the sequences corresponding to L1ASP (yellow block arrow), predicted open reading frames in the +2 (ORF 2A and ORF 2B, green block arrows) and +3 (ORF 3, red block arrow) frames, and endogenous polyA tract (double ended arrow). (**B**) Shown below is the sequence of LCT13a with the potential open reading frames (ORF) annotated. No putative conserved domains were detected by Blastp for any of the ORFs.

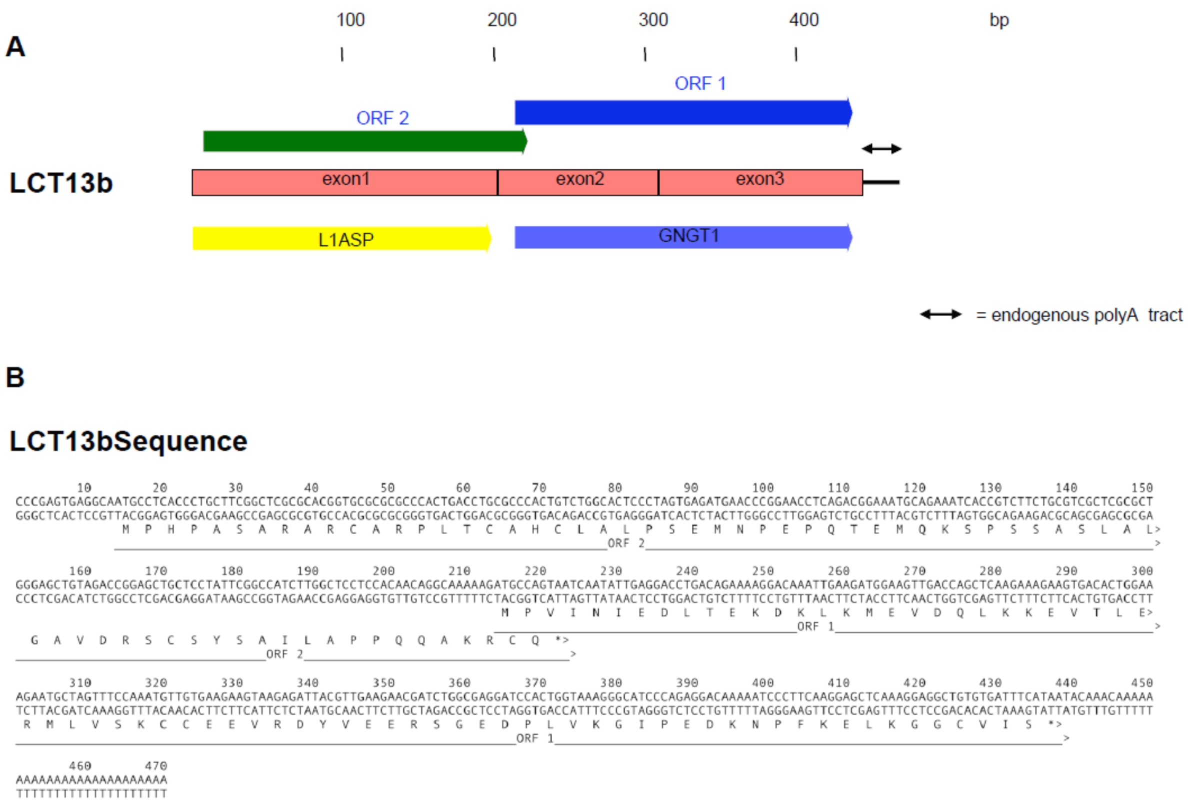

Supplementary Figure 4: The LCT13b transcript. **(A)** Schematic diagram depicting the exon structure of the LCT13b transcript with indicated the sequences corresponding to L1ASP (yellow block arrow) and *GNGT1* (blue block arrow below diagram). Predicted open reading frames in the +2 (ORF 2, green block arrow) and +1 (ORF 1, blue block arrow above diagram) frames and endogenous polyA tract (double ended arrow) are shown. (**B**) Below is the sequence of LCT13b with potential open reading frames (ORF). Given that the first exon of LCT13a and LCT13b is the same, ORF2 and ORF2A share the first 63 amino-acids and diverge at the C-terminus with ORF2A extending an additional 2 amino-acids in exon 2 of LCT13a and ORF2 extending 6aa in exon 2 of LCT13b. Similar to ORF 2A, no putative conserved domains were detected for ORF 2 by Blastp, whilst ORF 1 corresponds to GNGT1.

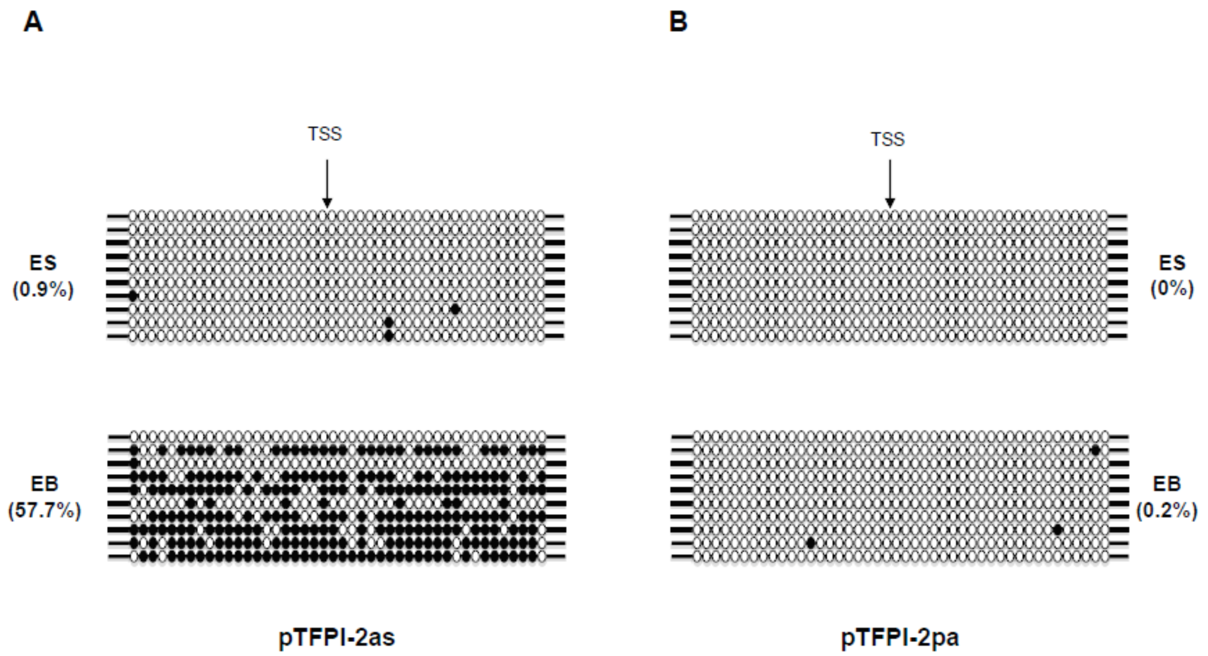

Supplementary Figure 5: Methylation of *TFPI-2* in differentiating pTFPI-2as and pTFPI-2pa ES cells. Bisulphite sequencing of *TFPI-2* CpG island DNA from in undifferentiated ES cells (ES) and day7 embryoid bodies (EB) containing pTFPI-2as (**A**) or pTFPI-2pa constructs. (**B**) In pTFPI-2as containing ES cells, which express an antisense transcript to TFPI-2, the *TFPI-2* gene is unmethylated (0.9%) however this becomes methylated upon differentiation to EB cells (57.7%). Cells containing the pTFPI-2pa construct, which do not express high levels of the antisense RNA, do not show DNA methylation in either ES or EB cells (0% and 0.2% respectively). TSS indicates the transcriptional start site of the *TFPI-2* gene; filled and open circle represent methylated and unmethylated CpG dinucleotides respectively. Each row represents an independent clone.

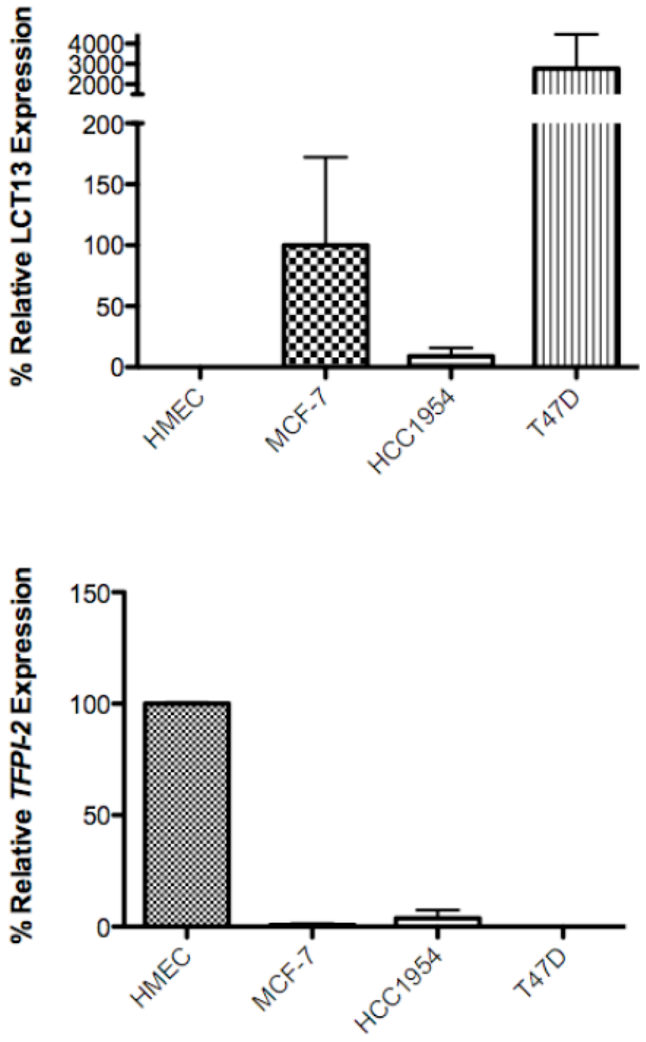

Supplementary Figure 6: Expression of LCT13b and *TFPI-2* in breast cancer cell lines. *TFPI-2* and LCT13b expression levels were measured by real time RT-PCR in total RNA from MCF-7, HCC1954 and T47D breast cancer cell lines and compared to HMEC cells as normal control. Values are normalised to *HPRT*.

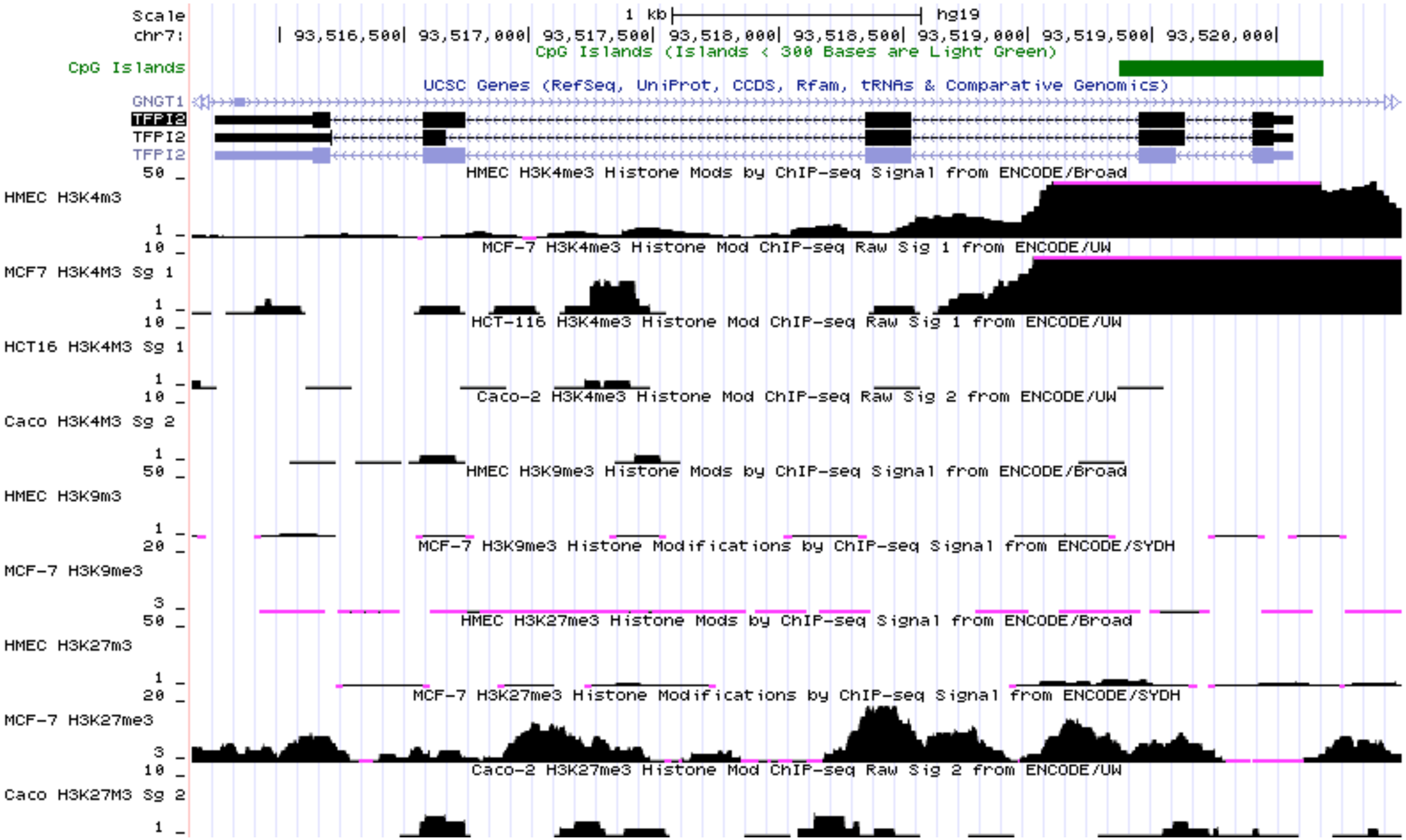

Supplementary Figure 7: ChIP data at the *TFPI-2* locus from ENCODE. USCS Genome browser snapshot of the *TFPI-2* gene with shown the currently available ENCODE ChIP-seq traces for histone modifications H3K4me3, H3K9me3 and H3K27me3 of HMEC, MCF-7, HCT116 (HCT16 traces) and CaCo2 (CaCo traces) cells. Note in particular the enrichment in both H3K4me3 and H3K27me3 at the *TFPI-2* promoter in MCF-7 cells confirming our ChIP findings (Fig. 5B and 7A). Traces for HCT116 H3K27me3 are not currently available.

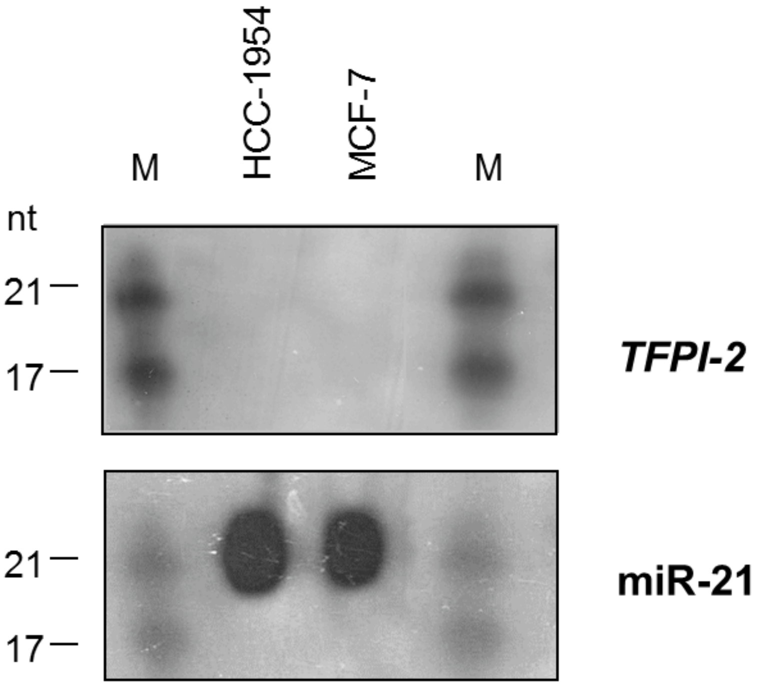

Supplementary Figure 8: Lack of detection of *TFPI-2* small RNAs in breast cancer cell lines. Size-fractionated, low molecular weight RNA from HCC-1954 and MCF-7 was analysed by northern blotting using a probe from *TFPI-2* CpG island sequence or the microRNA, miR-21 as a positive control for the detection of small RNA species. No small RNA species complementary to the *TFPI-2* promoter region were detected by this assay.

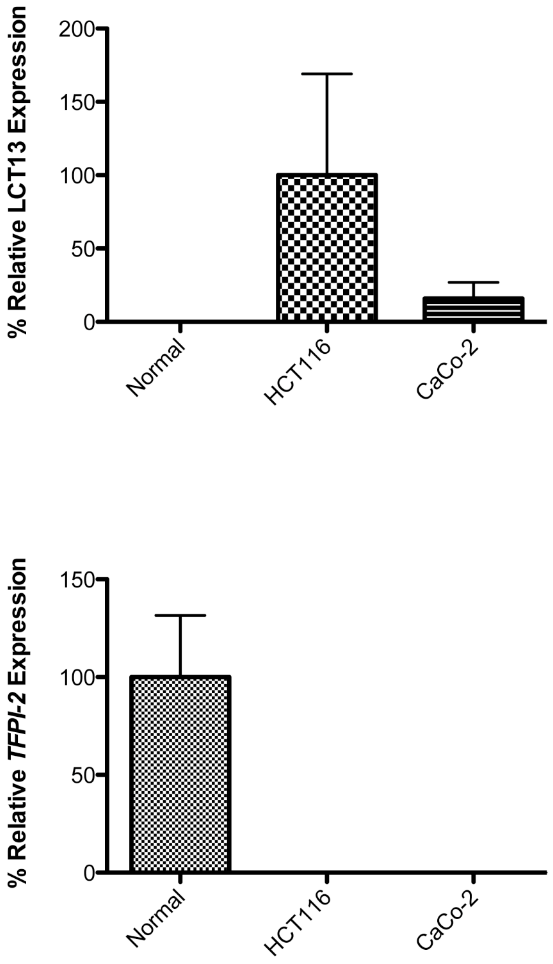

Supplementary Figure 9: Expression of LCT13b and *TFPI-2* in colon cancer cell lines. *TFPI-2* and LCT13b expression levels were measured by real time RT-PCR in total RNA from HCT116 and CaCo-2 colon cancer cell lines compared to normal colon. Values are normalized to *HPRT*.

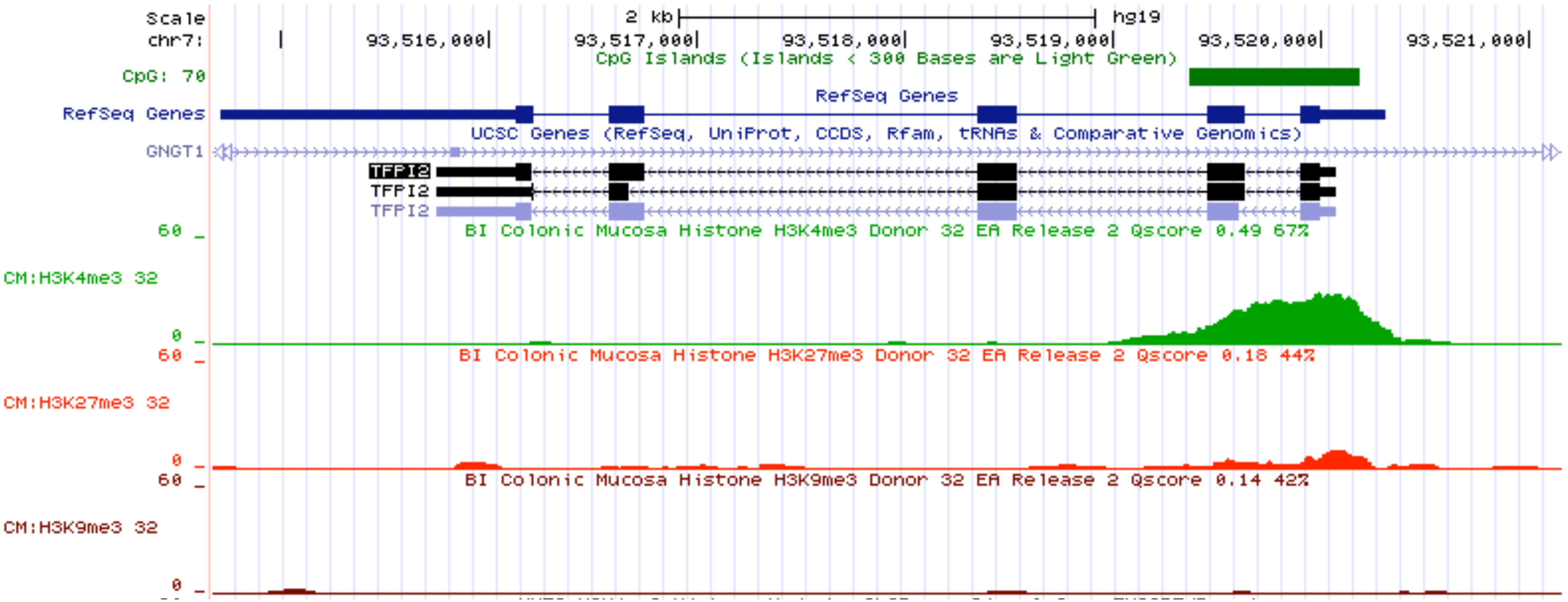

Supplementary Figure 10: ChIP data for normal colon mucosa generated by the Roadmap Epigenomics Project. UCSC genome browser snapshot of the *TFPI-2* locus showing tracks generated by chromatin immunoprecipitation of normal colon mucosa produced by the Roadmap Epigenome project and available from the Human Epigenome Atlas website (http://www.genboree.org/epigenomeatlas/multiGridViewerPublic.rhtml). Note that enrichment of H3K4me3 but very little or undetectable levels of H3K27me3 and H3K9me3 are present at the *TFPI-2* promoter in normal mucosa.

Supplementary Tables

Supplementary Table 1. List of primers used in strand specific reverse transcription

Primer name	Sequence (5'→3')	Target
CT51	CCAGCAAAGGAATGTGTTC	*APRT* 3'UTR
HC51c	CAATCCACGAGAGAAATGAGC	LCT13
HC58c	TACGCCTGACCACTTTCCCTC	TFPI-2as
HC64c	GTAAATGAATGGAGGATGATGGG	Region 3 in Figure 2A
HC65c	TTTGGGTCACAAGTTTCTGC	Region 4 in Figure 2A
HC66c	AATAGAGCAGGAATACAGAGGAC	Region 1 in Figure 2A
HC68c	GCAGTAAGATACGTGGTTATTGC	Region 5 in Figure 2A
HC69c	TGCTTGAAGGCACCATTAACTC	Region 6 in Figure 2A
Aprt 1	GGTAGCTCACAAAGGTCACTTAG	Mouse Aprt

Supplementary Table 2. PCR primer pairs

Primer name	Sequence (5'→3')[a]	Target	Annealing T (^{o}C)
CT49<br>CT50	TGGAGATTCAGAAAGACGCCC<br>GCCCTGTGGTCACTCATACTGC	*APRT*	57
HC66a<br>HC66b	CGTCTCTCTGAAATCTAATACACC<br>TCTTCAGTCATTTAGAATCATAGG	Region 1 in Figure 2A	54
HC20a<br>HC51b	AAATCACCGTCTTCTGCG<br>TGAGCAGTCTGGTTTATGGG	Region 2 in Figure 2A (LCT13)	51
HC64a<br>HC64b	CTTCACTGTAATGTGCCTGC<br>CCAACAGCAGTAAAGACACC	Region 3 in Figure 2A	51
HC65a<br>HC65b	TATGGCTAACAAAAGACCCTG<br>GAAGAGCCATTTCTGACACTG	Region 4 in Figure 2A	53
HC68a<br>HC68b	TGTCTATGACTTTGCTTATGCC<br>TAATCAAGGAGCTGGAGATAGG	Region 5 in Figure 2A	53
HC69a<br>HC69b	AGGTGACTGAGAGCATTGGG<br>AATCTAAATGTCCCCATCCTG	Region 6 in Figure 2A	54
HC58a<br>HC58b	GCACTTACTTTCTATCCTCCAGC<br>TCTCTTTTGCTCTCCTTCTGC	Region 7 in Figure 2A (TFPI-2as)	55
mAprt2<br>mAprt3	GGAAATCCAGAAAGATGCCTTGG<br>TCTAGCCAGCTCCTCAGTCATACTG	Mouse Aprt	60
HC63f<br>HC63g	CATTTACGG*GGTACC*TCTGCGACTTTGTCTTCTTGG<br>TCATTACGC*GGATCC*CTTTATTGGTGGCTACACAGTTG	Full length *TFPI-2*	55
Hind-p(A)-for<br>Hind-p(A)-rev	AATTCACCC*AAGCTT*CCTAAATGCTAGAGCTCGCTG<br>ACTAAGCCC*AAGCTT*CATAGAGCCCACCGCATC	BGH p(A)	55

[a] Bases in Italics correspond to restriction enzyme recognition sites added to primers for cloning purposes.

Supplementary Table 3. Primers used for amplification of bisulphite treated DNA

Primer name	Sequence (5'→3')	Annealing T (^{o}C)	Target (bp)
HC061a<br>HC061b	GGATTATAGGAGAAAGTTTGG<br>AAAAATTCTATCCCCTTCC	49	*TFPI-2* (540)
HC061c<br>HC061d	GAGAAAGTTTGGGAGGTAGG<br>CCTCTACAAAAAAAATACAAAC	48	*TFPI-2* (528)

Supplementary Table 4. Primers used with Roche multiprobe for real time PCR following ChIP

Primer name	Sequence (5'→3')	Target
HC074a<br>HC074b	CCCATTACTGACACAAACGC<br>CAAACTGTGTAAGAGGGAGAGG	*TFPI-2* prom
HC075a<br>HC075b	TAGAAAGTAAGTGCCCTGCG<br>TTCCTGTAGAAAGCGAGACG	*TFPI-2* ex-in2
ActBf<br>ActBr	TCAACACCCCAGCCATGTA<br>GTGGTACGACCAGAGGCATAC	Mouse beta actin

Supplementary Table 5. Taqman assays used in the study

Target	Sequence (5'→3') or inventory details	Size (bp)
LCT13b	Forward: GCTCCTATTCGGCCATCTTG<br>Reverse: TCCTTTTCTGTCAGGTCCTCAATAT<br>Probe: CTCCACAACAGGCAAA	81
TFPI-2prom	Forward: GGAATTCCCCGCCAAGTT<br>Reverse: CCGTCTGGACTACAGGAGAAAGTT<br>Probe: AAAAGTTGAACCTGCCTCC	63
TFPI-2 ex2	Forward: CTATCCTCCAGCAAGCATCGT<br>Reverse: GGGCAACGCCAACAATTTC<br>Probe: CAAGCCTCCCAGGTGT	58
APRT 3'UTR	Forward: GCCTCCCAGCCCAACATC<br>Reverse: GCAGTTGCCCAAGGCTGATA<br>Probe: CAGCTGGATCCCAGGGA	59
TFPI-2	ABI TFPI-2 assay: spans exon boundary 3- 4 (Hs00197918_m1)	84
GAPDH	ABI GAPDH assay: within exon9 (Hs03929097_g1)	58
HPRT	ABI HPRT assay: spans exon boundary 1-2 (Hs01003267_m1)	72
